# Nanomedical research and development in Spain: improving the treatment of diseases from the nanoscale

**DOI:** 10.3389/fbioe.2023.1191327

**Published:** 2023-07-21

**Authors:** Paula Fernández-Gómez, Carmen Pérez de la Lastra Aranda, Carlota Tosat-Bitrián, Jesús Alejandro Bueso de Barrio, Sebastián Thompson, Begoña Sot, Gorka Salas, Álvaro Somoza, Ana Espinosa, Milagros Castellanos, Valle Palomo

**Affiliations:** ^1^ Instituto Madrileño de Estudios Avanzados en Nanociencia (IMDEA Nanociencia), Madrid, Spain; ^2^ Centro de Investigaciones Biológicas Margarita Salas-CSIC, Madrid, Spain; ^3^ Centro de Investigación Biomédica en Red de Enfermedades Neurodegenerativas (CIBERNED), Instituto de Salud Carlos III, Madrid, Spain; ^4^ Centro de Investigaciones Energéticas, Medioambientales y Tecnológicas (CIEMAT), Unidad de Innovación Biomédica, Madrid, Spain; ^5^ Advanced Therapies Unit, Instituto de Investigación Sanitaria Fundación Jiménez Díaz (IIS-FJ UAM), Madrid, Spain; ^6^ Unidad Asociada al Centro Nacional de Biotecnología (CSIC), Madrid, Spain; ^7^ Instituto de Ciencia de Materiales de Madrid, ICMM-CSIC, Madrid, Spain

**Keywords:** nanoparticles, therapy, Spain, nanotechnology, nanomedicine

## Abstract

The new and unique possibilities that nanomaterials offer have greatly impacted biomedicine, from the treatment and diagnosis of diseases, to the specific and optimized delivery of therapeutic agents. Technological advances in the synthesis, characterization, standardization, and therapeutic performance of nanoparticles have enabled the approval of several nanomedicines and novel applications. Discoveries continue to rise exponentially in all disease areas, from cancer to neurodegenerative diseases. In Spain, there is a substantial net of researchers involved in the development of nanodiagnostics and nanomedicines. In this review, we summarize the state of the art of nanotechnology, focusing on nanoparticles, for the treatment of diseases in Spain (2017–2022), and give a perspective on the future trends and direction that nanomedicine research is taking.

## 1 Introduction

**FIGURE 1:**
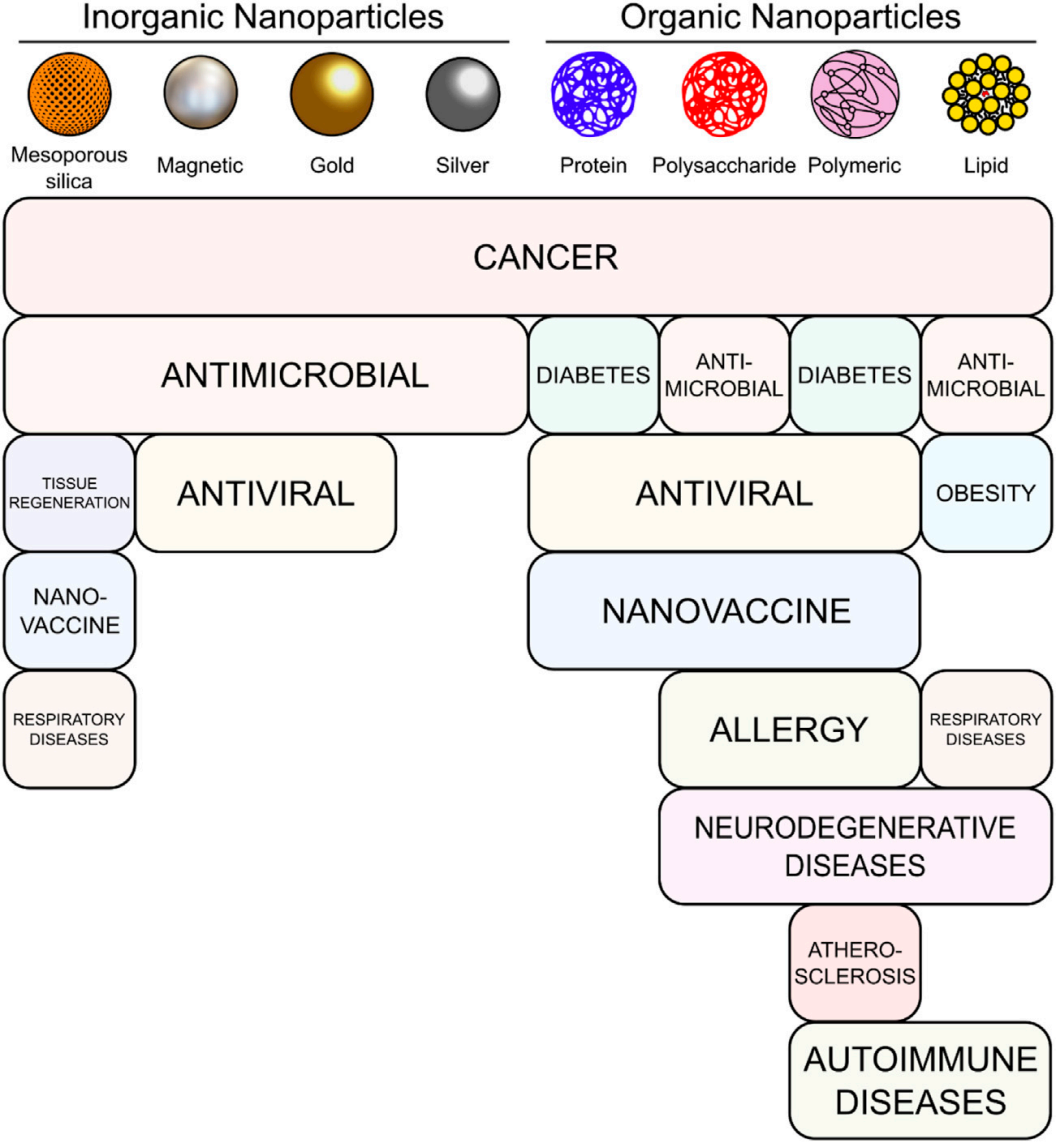
Main types of NPs and the therapeutic areas in which they are used in the articles included in this review.

Nanoparticles (NPs) are small particles usually around 10–100 nm in size, that can be obtained from a broad class of materials, (Khan et al., 2019), and are classified according to their nature (Figure 1). Materials have different properties at a nanometric scale, such as higher reactivity, singular optical or magnetic properties, among others. These properties can be used as warheads against pathological conditions (Fratila et al., 2019), (Guisasola et al., 2018a). Nanomedicine takes advantage of these unique features, offering a new set of therapeutic and diagnostic tools. For example, in NP-mediated hyperthermia, each NP acts as a heat source, increasing the temperature specifically at localized areas, damaging specific tumor cells in a controlled manner and reducing side effects in healthy tissues (in contrast with the bulk heating of conventional hyperthermia) (Vilaboa et al., 2017), (Sanz et al., 2017). A rise in temperature to 41°C–50°C induces cell death via necrosis and/or apoptosis, especially for the more thermosensitive cancer cells (Bass et al., 1978), (Sapareto and Dewey, 1984). In addition, hyperthermia increases blood irrigation preferentially within tumors (Elming et al., 2019), modifies the extracellular matrix of tumor tissue (Kolosnjaj-Tabi et al., 2017), and activates immunological responses by increasing the surface display of tumor antigens (Lee et al., 2018).

The shape, size, and surface of NPs are important properties to consider when using them for biological applications, since they determine the biocompatibility, biodistribution, cell-targeting, and uptake efficiency. NPs can be surface-modified with different biomolecules, including small proteins, antibodies, aptamers, oligonucleotides, oligosaccharides, polymers, or drugs. Therefore, by selecting suitable molecules, NPs can be tailored for the desired biological applications (Jindal, 2017), (Dolai et al., 2021), (Gatoo et al., 2014). In addition, the nanoformulation protects the cargo from degradation and improves its distribution in physiological media, facilitating oral administration and improving cell entry (Kim et al., 2021). Coating NPs with PEG, shields the surface from aggregation, opsonization, and phagocytosis, prolonging systemic circulation time and reducing their immunogenicity (Shi et al., 2021). Thus, different functionalization strategies make possible to enhance the pharmacokinetic properties of NPs, boosting the efficacy of therapy (Dacoba et al., 2017).

Another fundamental aspect is the protein corona formed on the surface of NPs, which plays a crucial role in the biological identity of NPs as it affects cytotoxicity, body distribution, endocytosis into specific cells, and biodegradation (Stepien et al., 2018), (Garcia-Alvarez and Vallet-Regi, 2021; Fleury et al., 2021). For these reasons, the proper identification and characterization of protein corona are essential in developing NPs-based therapeutics (Ritz et al., 2015; Di Silvio et al., 2018), (Alfranca et al., 2019).

NPs have a significant application as drug delivery systems (Miron-Barroso et al., 2021). Targeted therapies have at least three main advantages: reaching the target site specifically, not affecting other healthy organs, and reducing the dose needed to have the same therapeutic effect to the free drug. Biological barriers such as the blood-brain barrier (BBB) or mucus have a protective nature, hindering the simple diffusion of some therapeutic molecules, and a specific NP-mediated delivery can improve the permeability of therapeutic agents (Mulvihill et al., 2020), (Martin-Rapun et al., 2017). Finally, the possibility to encapsulate more than one drug that could provide synergistic effects showcases NPs versatility for delivery.

A significant example of the success of NPs as carriers is the drug Abraxane^®^. This nanomedicine, which comprises the chemotherapeutic paclitaxel (PTX) bound to albumin, has been approved for the treatment of metastatic breast cancer, advanced non-small cell lung cancer, late-stage pancreatic cancer, and metastatic triple-negative breast cancer. In several studies, it has increased patient survival and response rate significantly (De Luca et al., 2019).

Moreover, nanotechnology has also acquired a great interest in the immunological field. Vaccines are an extremely effective strategy to prevent several diseases, however their generation can be challenging since it is necessary to finely regulate the immunogenicity and the use of adjuvants as immunostimulatory agents can be critical to produce the desired effect. NPs have importance in vaccine generation because they are easily recognized by immune cells as they have a similar size to pathogens, and molecules can be anchored to their membrane to improve their recognition. In addition, new routes of administration, such as oral and nasal, can be used, and the possibility of multivalency enhances their activity (Miron-Barroso et al., 2021), (Gonzalez-Aramundiz et al., 2018).

Spain has emerged as a prominent player in the field of nanomedicine research in recent years. With numerous cutting-edge research centers and groups dedicated to this field, the country has made significant strides in the development of advanced treatments. Our review aims to provide a comprehensive overview of the latest breakthroughs in nanomedicine treatments in Spain over the past 5 years. By highlighting the capabilities of various research centers and groups, we hope to shed light on the role that Spain plays in advancing this exciting field.

## 2 Inorganic nanoparticles

Inorganic NPs encompass the nanoformulations that mainly contain inorganic elements. They include magnetic, metallic, gold, silver NPs and metallic quantum dots, among others, and have been studied as therapeutic systems against various diseases. For example, the plasmonic and optical properties of inorganic NPs have enabled to undertake innovative approaches to treat diseases, serving as improved contrast agents or thermo-photo-induced sources (Jaque et al., 2014), (Kim et al., 2018), (Borzenkov et al., 2019). Their tunable morphological properties and advantageous stability have promoted great promises in their nanomedical use. However, the long-term effects of the administration of these NPs in the human body still needs exhaustive characterization, which has resulted in a slower clinical translation of these NPs in comparison to their organic counterparts (Paul et al., 2020). In this review, recent advances in silica, magnetic, gold, and silver NPs, are summarized. At the end of each section, the studies are summarized in tables and categorized by NP type highlighting their purpose, therapeutic area, agent and functionalization strategy.

### 2.1 Mesoporous silica nanoparticles

Mesoporous silica NPs (MSNs) present several advantages that have qualified them as ideal carriers for drug delivery (Manzano and Vallet-Regi, 2019), (Manzano and Vallet-Regi, 2018), (Iturrioz-Rodriguez et al., 2019). They have large pores (0.6–1 cm^3^) to encapsulate molecules of different sizes (including proteins), an easily modifiable surface that allows controlling drug loading and release, a very high surface-to-volume ratio, and good biocompatibility (Villegas et al., 2018), (Vallet-Regi et al., 2022). In addition, they can be prepared at different sizes (50–200 nm) and present a large surface area (Vallet-Regi et al., 2017). The group of Vallet-Regí was the first one to report the use of ordered mesoporous silica for drug delivery using the mesoporous material MCM-41 for the controlled release of ibuprofen using a simulated body fluid (Vallet-Regi et al., 2001). After that, tailored strategies have been developed for an efficient and smart delivery of therapeutic molecules, mainly for cancer treatment. For example, MSNs can be tuned to be responsive to a specific pH through an acid-sensitive linker, increasing tumor selectivity and efficacy, (Martinez-Carmona et al., 2018), and they can also be tuned to target specific organelles (Table 1) (Gisbert-Garzaran et al., 2020).

**TABLE 1:** Summary of MSNs with their therapeutic area and functionalization strategy.

NP Type	Purpose	Therapeutic area	Therapeutic agent	Functionalization strategy	Ref.
Gated MSNs	Delivery	Antimicrobial	Essential Oils	Lactose as molecular gate	Poyatos-Racionero et al. (2021)
Multifunctional MSNs		Cancer	DOX	Layer by layer functionalization with benzimidazole and β-cyclodextrin gold NPs as pH sensitive gate	Jimenez-Falcao et al. (2019)
MSNs				Galacto-oligosaccharides covered MSNs to target senescent tumoral cells	Munoz-Espin et al. (2018)
Nanovehicle MSNs			Camptothecin and DOX	pH responsive PEG linker	Llinas et al. (2018)
MCM-41/Pt			Ru(Bpy)_3_Cl_2_/DOX	Catalytic self-propulsion and oligoethylenglycol containing a disulfide element as redox dependent gating system	Diez et al. (2021)
TNFR-Dex-MSN		Pulmonary diseases	Dexamethasone	Functionalized with a peptide targeting TNFR1 that avoids cargo release. After internalization and peptide hydrolysis, the cargo is released	Garcia-Fernandez et al. (2021a), Garcia-Fernandez et al. (2021b)
Monodisperse silica spheres		-	-	Particles were functionalized with carbon nanotubes and linked to fluorophores	Iturrioz-Rodriguez et al. (2017)
Au-MSNs Janus	Development of an enzyme-controlled NP to release DOX in cancer cells	Cancer	DOX	MSNs capped with a thiol sensitive gate and glutathione reductase on the gold face, that controls cargo release	Mayol et al. (2021)
MSN loaded with iron oxide	Synergy of hyperthermia and drug delivery			SPIONS in MSNs covered with small PEG chains and a shell of polymer sentitive to temperature	Guisasola et al. (2018b)
Janus MSNs	Improve controlled targeting and current nanomedicines		Topotecan	Asymmetrically functionalized with two targeting moieties, folic acid and triphenylphosphine to target tumor cells and mitochondria respectively	Lopez et al. (2017)
MSNs	Overcoming biofilm barrier	Antibacterial	Levofloxacin	Concanavalin A was attached to carboxylic groups grafted on the MSNs surface forming covalent amide bonds	Martinez-Carmona et al. (2019)
AuNR@MSNs-SNO	Develop NIR activated MSNs combinade with photothermal and antimicrobial ccapabilities			Gold nanorods were covered in a silica shell functionalized with PEG and thiol groups to be attached to tert-butylnitrite	García et al. (2021)
MSNs	Induce biofilm disaggregation		Moxifloxacin, rifampicin	Gelatin/colistin coated MSNs to avoid premature antibiotic release	Aguilera-Correa et al. (2022a)
MSNs-AgBr and AG@MSNs	Improve AgNPs effect	Tuberculosis	-	-	Montalvo-Quiros et al. (2021)
MSNs@PEI	Silence genes to stimulate bone regeneration	Bone regeneration	Osteostatin	Polyethylenimine grafted MSNs on phosphonate-modified MSNs	Mora-Raimundo et al. (2019)
MSNs	Induce osteogenesis and bone repair		Ipriflavone	Spherical NPs with a porous core-shell structure synthesized by double template method	Arcos et al. (2022)
Mesoporous nanospheres	Promote vascularization	Tissue regeneration		-	Casarrubios et al. (2021)
	Study NPs effects on osteoprogenitor cells	Periodontal		-	Casarrubios et al. (2020)
MSNs	Vaccine development	Tuberculosis	-	MSNs loaded with immunomodulatory proteins	Montalvo-Quiros et al. (2020)
SiO_2_@ShTxB:FITC and Fe_3_O_4_@SiO_2_:RBITC@ShTxB		Head and Neck Cancer	-	Particles were functionalized with fluorophores and with the protein through sonication methodology	Navarro-Palomares et al. (2021)

The delivery of the specific agent of MSNs can be controlled in different manners (Vallet-Regi et al., 2022). For example, Poyatos-Racionero et al. prepared MSNs loaded with essential oil components and covered by lactose that functioned as a molecular gate. They explored the properties of these particles loaded with different active agents in cellular and animal models, confirming the potential of this strategy for controlled delivery (Poyatos-Racionero et al., 2021). With regard to toxicity, mesoporous silica rods (MSR) have been less investigated in terms of biodistribution, biocompatibility, and cellular uptake, however in this case they presented improved characteristics when compared to their spherical counterparts in animal models. In this scenario, MSRs were functionalized with magnetic and fluorescent elements for diagnosis and treatment of fibrotic liver diseases (Grzelak et al., 2022).

Jimenez-Falcao et al. brought further the strategy of on-command delivery engineering loaded MSNs with a layer-by-layer supramolecular architecture, each with a specific role. The particles were first functionalized by benzimidazole and β-cyclodextrin gold NPs that act as a pH-sensitive gate. Then, a final coating was performed with glucose oxidase modified with an adamantine moiety linked to the free cyclodextrins. In that manner, these conjugates delivered their cargo upon the addition of glucose and were able to reduce HeLa cell viability (Jimenez-Falcao et al., 2019). A different delivery strategy was developed by Muñoz-Espín et al. to target senescent cells. They used MSNs coated with galacto-oligosaccharides, taking advantage of the high activity of lysosomal β-galactosidase activity in senescent cells. The nanoconjugates showed a preferential accumulation in senescent cells in animal models, improving tumor regression in mice and reducing the side effect of toxic drugs (Munoz-Espin et al., 2018).

To enhance delivery and exploit different ways of cellular entry, Navarro-Palomares et al. took advantage of cytoplasmatic entry of the toxic Shiga protein. They prepared fluorescent MSNs conjugated to a safe fragment of the protein that enabled to deliver the NPs intracellularly by a non-canonical pathway and thus avoiding the endolysosomal entry and its associated degradation (Navarro-Palomares et al., 2021).

Llinas et al. prepared a pH responsive nanosystem to deliver several drugs. First, they developed a nanosystem capable of delivering camptothecin (CPT) and doxorubicin (DOX) (Llinas et al., 2018), and, in a further step, they developed a new system for the delivery of CPT, DOX, and zinc (II) phthalocyanine (Pc). They labelled (Pc-CPT)@MSN-hyd-PEG-hyd-DOX, which sequentially releases DOX, linked on the MSNs surface through a pH-sensitive PEG linker that gradually delivers the Pc-CPT conjugate loaded inside the MSNs. In this manner, they combined chemotherapy and photosensitizers for photodynamic therapy (PDT). Upon irradiation of the samples, the Pc phototoxicity enhances the chemotoxicity of DOX and CPT (Martinez-Edo et al., 2021).

**FIGURE 2:**
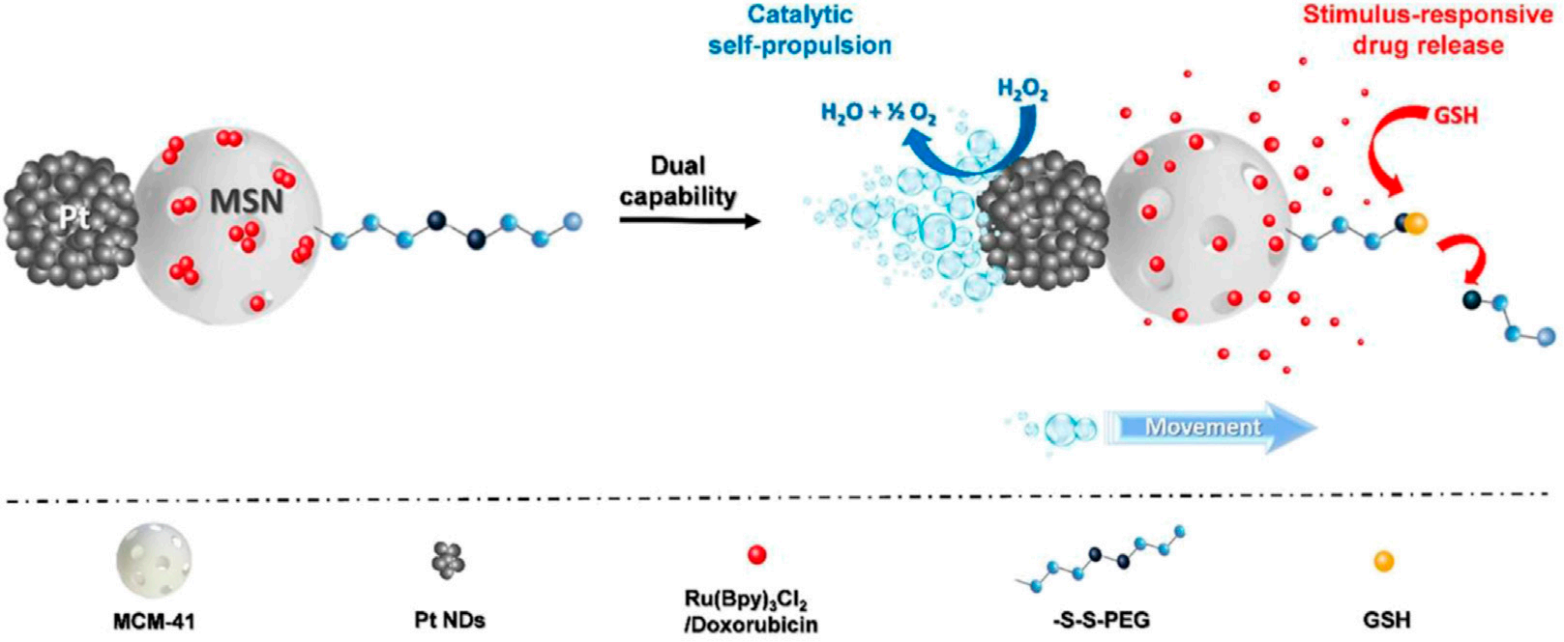
Design of Janus Pt-MSN motors. Reprinted from Diez et al. (Diez et al., 2021), and licensed under the Creative Commons Attribution.

In a further step, self-propelled NPs also enable a more efficient manner to reach their target site without the need for external stimuli. Diez et al. designed MSNs coupled with platinum nanodendrites as a self-propulsion element (Figure 2). The particles were designed with an oligoethylenglycol containing a disulfide element that acts as a gating system that can be opened under specific redox conditions. This is a successful proof of concept of a nanomaterial that can autonomously reach its target and deliver the cargo upon specific and controllable conditions (Diez et al., 2021).

Another complicated site for drug delivery are the lungs, which present several pulmonary barriers that prevent the effective delivery of traditional drugs. In this regard, García-Fernández et al. addressed this issue using MSNs (Garcia-Fernandez et al., 2021a), (Garcia-Fernandez et al., 2021b). In this case, the nanostructures were loaded with dexamethasone, which is the standard corticoid used for the treatment of this disease, and decorated with a peptide with a dual objective, targeting TNFR1 receptors and avoiding the release of the cargo. TNFR1 receptors are expressed in pro-inflammatory macrophages, and indeed the particles showed a selective uptake by these cells and released the drug. The conjugates were also effective in animal models, demonstrating lung accumulation and the reduction of the damage.

As seen by now, the versatility of NP development with therapeutic approaches enables infinite combinations of coatings, particle size, shape, and biomolecule incorporation. In addition, one can combine several NPs into new assemblies that enable to take advantage of several properties simultaneously (Redolfi Riva et al., 2017). When NPs display two or more physical properties divided on their surface, they are called Janus particles, due to their asymmetric geometry. They can also be prepared with the combination of different kinds of NPs that enable them to take advantage of both materials, and even unlock undiscovered synergistic effects. Mayo et al. developed mesoporous silica-gold Janus NPs linked to glutathione reductase. These assemblies resulted highly efficient in delivering their cargo when presented with NADPH and glutathione disulfide as triggers (Mayol et al., 2021).

Similarly, Guisasola et al. developed innovative MSNs loaded with iron oxide NPs coated with a polymer sensitive to temperature. Upon the application of alternating magnetic fields (AMF), DOX was effectively released in animal models without increasing global tissue temperature, provoking a synergistic effect of drug and hyperthermia antitumoral activities (Guisasola et al., 2018b). In another example of Janus-type MSNs, Lopez et al. designed particles selective for tumor cells that, once internalized, specifically targeted mitochondria organelles, showcasing the utility of this strategy (Lopez et al., 2017).

Bacterial resistance to common antimicrobials is growing, and many efforts are being dedicated to the development of new antibiotic tools. Biofilm formation is especially problematic because it requires much higher antibiotic doses. MSNs can be designed to deliver antimicrobials in a localized and efficient way (Bernardos et al., 2019), (Vallet-Regi et al., 2019), (Colilla and Vallet-Regi, 2020). This approach has been used in MSNs functionalized with concanavalin A, which promotes the internalization of the NPs into the biofilm matrix to deliver levofloxacin (Martinez-Carmona et al., 2019). Also, in a sophisticated example, the release of levofloxacin and nitric oxide in biofilms was enhanced by near-infrared (NIR) irradiation using core-shell Au-MSN NPs (García et al., 2021). Aguilera-Correa et al. developed gelatin/colistin coated MSNs to treat osteomyelitis, a bone infection with poor prognosis. The functional coating prevented premature antibiotic release and induced biofilm disaggregation, showing the potential of these NPs to treat bone infections (Aguilera-Correa et al., 2022a). Finally, Montalvo-Quirós et al. explored the antimicrobial activity of MSNs loaded with silver bromide NPs and silver NPs with a mesoporous silica shell, confirming the great potential of MSNs for this application (Montalvo-Quiros et al., 2021).

Another prominent field of nanomedicine is the use of MSNs in bone regeneration. Mora-Raimundo et al. engineered particles to simultaneously deliver siRNA, to silence genes that inhibit osteoblasts differentiation, and osteostatin, which stimulates bone regeneration in animal models, taking advantage of the high loading capacity of MSNs (Mora-Raimundo et al., 2019). Further, Arcos et al. developed a methodology to inject MSNs loaded with an antiosteoporotic drug, ipriflavone, for the first time in rabbits, by suspending them in a hyaluronic acid hydrogel. The particles induced osteogenesis and bone repair (Arcos et al., 2022). In the same field, Casarrubios et al. decided to take advantage of the essential role that angiogenesis plays in vascularization and tissue regeneration. They loaded mesoporous nanospheres with ipriflavone, showing its release in endothelial cells by the increase of VEGFR2 expression indicating angiogenesis (Casarrubios et al., 2021). In another publication, the authors explored the potential of ipriflavone MSNs for periodontal treatment. They confirmed the clathrin-mediated entrance of the NPs, showing an osteogenesis activity (Casarrubios et al., 2020).

An original manner to deliver NPs was developed by Iturrioz-Rodriguez et al. that coated silica NPs with carbon nanotubes that enabled a cytoplasmic delivery and opens an innovative pathway to reach the cytoplasm of cells (Iturrioz-Rodriguez et al., 2017).

Finally, MSNs have also ideal properties to become a platform for vaccine development. Montalvo-Quirós et al. engineered MSNs loaded with immunomodulatory proteins that showed to have protective effects against infection of tuberculosis, (Montalvo-Quiros et al., 2020), and that could be used for dual delivery of immunomodulatory proteins and antitubercular drugs.

### 2.2 Magnetic nanoparticles

Magnetic NPs (MNPs) are an exceptional tool for biomedical treatment. They can deeply internalize in tissues, and have magnetic-heating capability. The most explored MNPs for nanomedicine are, by far, superparamagnetic iron oxide NPs (SPIONs) and related ferrites (Roca et al., 2019), (Pardo et al., 2020), (Mazario et al., 2012), (Garcia-Soriano et al., 2020), (Rubia-Rodriguez et al., 2021a). In clinical practice and biomedical research, they are used as iron supplements, contrast agents, and magnetic hyperthermia therapeutics (Polo et al., 2018), (de la Presa et al., 2012). Through the later application, localized heat is generated, which increases gene expression (Moros et al., 2019), particularly of the heat shock protein family, and the formation of reactive oxygen species (ROS), while inducing apoptosis and several cellular stresses such as endoplasmic reticular stress or mitochondrial damage. Interestingly, cancer cells are more sensitive to heat than healthy ones, and therefore, this approach can present reduced toxicity in healthy cells and tissues. On the other hand, the requirement to use this therapy is the application of an alternating magnetic field (AMF), which has a high penetration and negligible effect on the tissues, compared with other antitumoral techniques that present toxic side effects for nearby healthy cells and tissues. Finally, magnetic hyperthermia can be used to promote drug release and therefore it can be used alone or in combination with drugs, for synergistic combinatory therapy, or with specific molecules for active targeting (Table 2). For example, Fe_3_O_4_ NPs were synthesized by a seed growth method with defined shapes and sizes and were functionalized with an arginine-glycine-aspartate (RGD)-type peptide to target αvβ3 integrin receptors over-expressed in angiogenic cancer cells. NPs showed a good heating response, lower toxicity and better biocompatibility with improved magnetic properties (Arriortua et al., 2018). Sanz et al. extensively studied and compared conventional hyperthermia and the therapeutic advantage of using MNPs, confirming the improved effectiveness of the nanoheaters (Sanz et al., 2017). In case of other type of hyperthermia, Lozano-Pedraza et al. explored the optical heat losses using iron oxide NPs and identified the parameters that influence the NIR-heating effects for therapeutic purposes (Lozano-Pedraza et al., 2021).

**TABLE 2:** Summary of MNPs with their therapeutic area and functionalization strategy.

NP Type	Purpose	Therapeutic area	Therapeutic agent	Functionalization strategy	Ref.
Iron oxide (magnetite/maghemite)	Study of cell internalization and effects of IONPs	Cancer	-	Citric acid-coated IONPs	Cabrera et al. (2018)
	MHT: cytotoxicity study		-	3-aminopropyl-triethoxysilane and dimercaptosuccinic acid coated MNPs	Mejias et al. (2019)
	MHT control		DOX	Silica coated MNPs functionalized with hydroxyl groups	Fuentes-García et al. (2021)
	MHT with 3D cultures		-	MNPs coated with poly(maleic anhydride-alt-1-octadecene) modified with TAMRA functionalized with glucose	(Beola et al., 2018), (Beola et al., 2020)
	MHT: conditions for improving treatment effectiveness *in vivo*	Pancreatic cancer	-	MNPs coated with poly(maleic anhydride-alt-1-octadecene) modified with carboxytetramethylrhodamine functionalized with glucose	Beola et al. (2021)
	Multi-Hot-Spot magnetic inductive nanoheating	Selective regulation of multienzymatic reactions	-	Dimercaptosuccinic acid, poly(maleic anhydride-alt-1-octadecene) or poly(acrylic acid) coated MNPs	Ovejero et al. (2021b)
	MHT in mice. Study on the reliability of NP synthesis and how to control T increase	Pancreatic cancer	-	Dextran or starch coated MNPs	Luengo et al. (2022)
	MHT: chemotherapeutic synergy with drug delivery	Cancer	Gemcitabine	N6L ligand and gemcitabine functionalized on albumin coated MNPs	Aires et al. (2017)
	MHT and chemotherapeutic drug nanocarriers	Breast cancer	DOX	Dimercaptosuccinic acid coated MNPs functionalized with DOX using three different linkers-disulfide, imine or both	Lazaro-Carrillo et al. (2020)
	Selective multimodal treatment of pancreatic cancer	Pancreatic cancer	Gemcitabine	Dimercaptosuccinic acid coated MNPs functionalized with Gemcitabine and anti-CD47 antibody	Trabulo et al. (2017)
	Antiproliferative properties	Cancer	Crocin	Dextran and crocin coated MNPs	Saravani et al. (2020)
	Cell retention to improve cell therapy, EMF	Cell therapy in cancer	-	Dimercaptosuccinic acid, 3-aminopropyl-triethoxysilane or dextran coated MNPs	Sanz-Ortega et al. (2019a)
	Adoptive T cell-transfer, EMF[Fn fn2]		Antibodies	3-aminopropyl-triethoxysilane coated MNPs functionalized with antibodies	Sanz-Ortega et al. (2019b)
	Improve adoptive cell transfer therapy, EMF		-	Dimercaptosuccinic acid, 3-aminopropyl-triethoxysilane or dextran coated MNPs	Sanz-Ortega et al. (2019c)
	Gene transfection, pro-inflammation, magnetic targeting and anti-angiogenesis	Cancer	Polyethylenimine	Polyethylenimine-coated SPIONs	Mulens-Arias et al. (2019)
	Smart miRNA delivery system for immunotherapy		miRNA155, miRNA125b and miRNA146a	Dextran, carboxymethyldextran or dimercaptosuccinic acid coated NPs	Lafuente-Gomez et al. (2022)
Iron oxide (magnetite/maghemite)	PTT using iron oxide NPs	Cancer	-	IONPs with different sizes and coatings for biocompatibility	Lozano-Pedraza et al. (2021)
	PEI-MNPs	Cancer	-	Polymer coated NPs (PEI)	Sanz et al. (2017)
Iron oxide (magnetite) NPs and iron oxyhydroxide NPs	Prophylactic or therapeutic treatments for SARS-CoV-2	SARS-CoV-2	-	Dimercaptosuccinic acid, 3-aminopropyl-triethoxysilane or carboxydextran coated IONPs and sucrose coated iron oxyhydroxy NPs	DeDiego et al. (2022)
Ferrites M_x_Fe_3-x_O_4_ (M = metal other than iron)	Develop microswimmers with MHT capacity	Sarcoma (collagen-rich ECM)	-	PLL and collagenase coated polystyrene particles containing manganese ferrite NPs	Ramos-Docampo et al. (2019)
	Describe MHT and NPs uptake in cancer cells	Glioblastoma	-	cRGD peptide conjugation in dimercaptosuccinic acid manganese ferrite NPs	Del Sol-Fernandez et al. (2019)
	Promote heterogeneous catalysis for ROS production	Cancer	-	Copper-iron oxide spinel NPs. BSA templated synthesis with ethylene glycol	Bonet-Aleta et al. (2022a), Bonet-Aleta et al. (2022b)
Hybrid magnetic nanomaterials^1^					
Iron oxide-MnO_2_	Intracellular response for switchable MRI contrast and magnetic hyperthermia	Cancer	-	Core-shell NPs with a tunable Mn oxide shell growth over iron oxide NPs	Garcia-Soriano et al. (2022)
Iron oxide-gold	Multifunctional photothermal therapy (PTT)		-	Gold suprashells around dextran coated SPIONS	Paterson et al. (2017)
	Multimodal cancer theranostics		-	Iron oxide-gold nanoflowers with PEGylated ligand	Christou et al. (2022)
	PTT, MHT and magneto-photothermal treatment with Janus NPs		-	Polyvinylpyrrolidone-coated iron oxide-gold magnetic Janus nanostars	Espinosa et al. (2020)
	PTT, MHT and magneto-photothermal treatment		-	Au-coated rod-shaped magnetite NPs in agarose hydrogels	Rincon-Iglesias et al. (2022)
Iron oxide-silver	Synergy between Ag and MHT	Antibacterial	-	γ-Fe_2_O_3_-Ag nanocomposites	Luengo et al. (2020)
	MHT *in vivo* real time feedback	Cancer	-	Phospholipid encapsulated Iron oxide-Ag_2_S nanocomposites	Ximendes et al. (2021)

^1^ Hybrid here refers to nanostructures combining two types of inorganic materials in which both are clearly distinguishable (e.g., core-shell, aggregates/encapsulates of individual NPs of both materials) and both provide a relevant function for the application (i.e., are not used just as a coating or as a platform).

**FIGURE 3:**
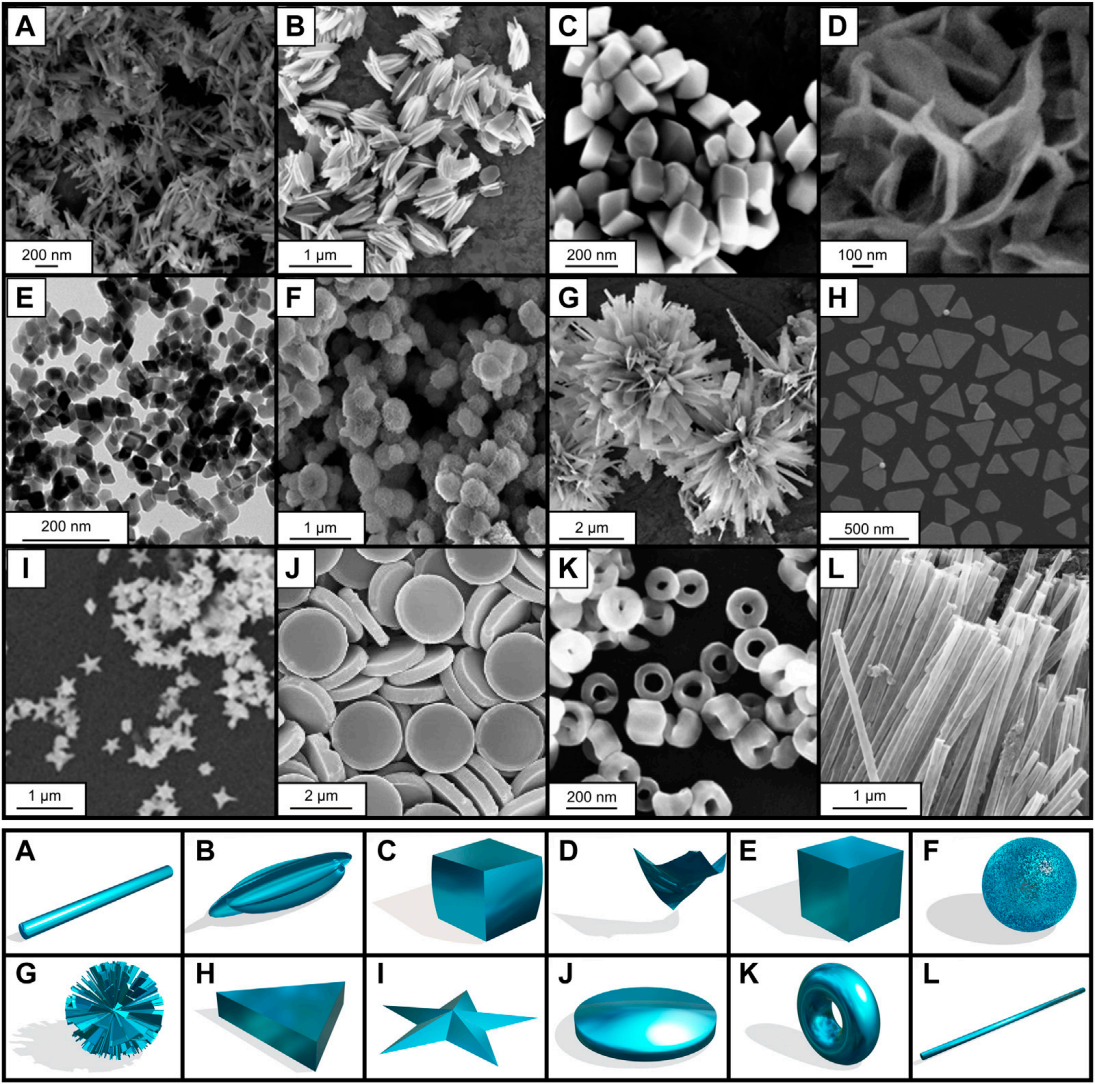
Different shapes and morphologies of iron oxide and other NPs. Scanning and transmission electron microscopy images on the upper part and morphology representation on the lower part. **(A)** nanorod, **(B)** nanohusk, **(C)** distorted cubes, **(D)** nanosheets, **(E)** distorted cubes, **(F)** porous spheres, **(G)** self-oriented flowers, **(H)** prismatic IONPs, **(I)** nanostar, **(J)** nanodiscs, **(K)** nanorings and **(L)** nanotubes. **(A, B, C, E, F, G)** adapted from Sayed and Polshettiwar (2015), **(D)** adapted from Chin et al. (2007), **(I)** adapted from Becerril-Castro et al. (2022), **(J)** adapted from Qu et al. (2021), **(H)** adapted from Ramirez-Jimenez et al. (2020), **(K)** adapted from Jia et al. (2008), **(L)** adapted from Zhang et al. (2016). Licensed under a Creative Commons Attribution.

Remarkably, iron oxide and other NPs can be obtained in a wide variety of morphologies and anisometric properties (Figure 3) (Roca et al., 2019) including, rods, cubes, stars, rings or as flower-shaped NPs among others (Gavilán et al., 2017). It is worth mentioning that the biological properties (e.g., internalization, toxicity) and response to AMF vary upon specific morphology (Ovejero et al., 2021a), (Simeonidis et al., 2016).

MNPs can be also combined with other nanostructures to yield nanostructures with additional properties. In this regard, Paterson et al. engineered self-assembled gold suprashells around dextran-coated SPION cores, which allowed them to obtain nanostructures with plasmonic and magnetic properties. The use of magnetic fields can be used to promote the accumulation of these nanostructures in cancer cells, and then exploit the plasmonic properties to induce heat by a light source, leading to the death of the tumoral cells (Paterson et al., 2017). In another work, MNPs were functionalized with dextran and crocin, which has antiproliferative properties. Crocin-coated dextran-MNPs showed greater anti-tumor effects and a higher rate of early apoptosis on MCF-7 breast tumor cells than free crocin was obtained, suggesting an effective alternative to traditional cancer treatments (Saravani et al., 2020).

Mejias et al., have worked in hyperthermia that improves tumor antigen presentation, activation of dendritic cells and natural killer cells, and leukocyte trafficking through endothelium. Probably due to NP aggregation, the contact of MNPs with cells could affect the heating capacity, highlighting the importance of NP coating to avoid cell-induced aggregation (Mejias et al., 2019). Similarly, Cabrera et al. studied intracellular MNP clustering, that led to a reduction of the magnetic hyperthermia ability. This work allowed to predict the magnetic thermal response of several NPs sizes in the cellular media (Cabrera et al., 2018).

Beola et al. investigated MNPs activity in 3D cell cultures, showing that magnetic hyperthermia can trigger necrosis or disruption of the extracellular matrix depending if the MNPs are inside or outside the cells (Beola et al., 2018). They studied the cell death mechanisms and the influence of the number of internalized particles to the cytotoxic effect testing several concentrations up to 7.5 pg Fe/cell. They showed that different apoptotic routes are triggered depending on the number of internalized NPs (Beola et al., 2020). In another work, they selected the conditions that caused the largest effect in cell viability for testing the NPs in animal models (Beola et al., 2021). AMF promoted MNPs migration into the tumor and confirmed that NP biodistribution is essential for hyperthermia effectiveness, and is affected by surface coating, playing the protein corona a significant role (Stepien et al., 2018).

Also employing 3D cell cultures, the group of V. Salgueiriño and co-workers described the assembly of magnetic microswimmers, composed of 500 nm polystyrene particles containing ferrite NPs. The motion of the self-propelled microswimmers was triggered by calcium, and they were able to penetrate spheroid models for heat delivery under AMF (Ramos-Docampo et al., 2019).

Luengo et al. synthesized maghemite NPs with different coatings to determine the best properties to use in clinical applications. The NPs were injected into animals with pancreatic cancer, and the results determined that modulating the field intensity can control the temperature rise during magnetic hyperthermia protocols in animal models (Luengo et al., 2022).

The combination of experimental and simulation approaches might be a useful tool for better engineering NPs. In this regard, a model has provided quantitative predictions to fit the properties of iron NPs, including a targeting agent and a drug. Particularly, it allowed the design of NPs with a pseudopeptide Nucant-6L, which induced a significant accumulation in tumors. The studies revealed the synergy of Nucant-6L, the chemotherapeutic drug gemcitabine, and the NPs, together with the importance of fine tuning the functionalization (Aires et al., 2017).

Christou et al. developed a seed-assisted methodology for the synthesis of gold and iron oxide nanoflowers. The particles were functionalized with PEG, greatly enhancing the colloidal stability of the conjugates. The nanoflowers performed highly as contrast agents and exhibited a considerable conversion of energy to heat, having ideal properties to be used as theragnostic agents (Christou et al., 2022).

Del Sol-Fernandez et al. also developed flowerlike manganese iron oxide cRGD-functionalized NPs that, when exposed to the appropriate AMF conditions, induced intracellular magnetic hyperthermia resulting in *hsp70* transcription and strong ROS production leading to cell death in a glioblastoma cell line (Del Sol-Fernandez et al., 2019).

Espinosa et al. developed Janus magneto-plasmonic NPs, using gold nanostars and iron oxide nanospheres subjected to an external magnetic field and NIR light. With this strategy, a synergistic cytotoxic effect on cancer cells was achieved based on the combination of the two thermal effects into a magneto-photothermal modality. Moreover, experiments in animal models confirmed the high efficiency of magnetically enhanced photothermal therapy (PTT) that led to tumor growth inhibition, and the delivery was highly improved by magnetic targeting (Espinosa et al., 2020). Another type of magneto-plasmonic materials that display magneto- and photothermal anisotropic transductions for cancer ablation has been proposed by Rincon- Iglesias et al., that incorporated Fe_3_O_4_@Au nanorods in an agarose hydrogel, resulting in free-standing anisotropic materials (Rincon-Iglesias et al., 2022).

Mulens-Arias et al., investigated the modulation of angiogenesis as an antitumor therapy. They used MNPs and a magnetic field for this approach. PEI-SPIONs, (SPIONs coated with polyethylenimine) showed anti-angiogenic and antitumoral effects as these NPs were able to reduce tumor vessel numbers and promoted intratumor macrophage infiltration in a tumor model after administration and application of magnetic field (Mulens-Arias et al., 2019). As another strategy against cancer, Sanz Ortega et al. developed NPs-based drug delivery systems to increase immunotherapy effectiveness. They showed that MNPs and the use of AMF can guide and retain T lymphocytes to a target region of interest and can be magnetically retained there (Sanz-Ortega et al., 2019a), (Sanz-Ortega et al., 2019b). In addition, they took advantage of the role of natural killer cells in antitumor immunity by binding MNPs coated with 3-aminopropyl triethoxysilane (APTES) to the surface of natural killer cells. They reported the retention of the cells at the specific target site by using external magnetic fields as the magnetic guiding effect (Sanz-Ortega et al., 2019c).

While hyperthermia has been exploited using several conjugates, an unsolved problem in this field is the lack of real time information on the temperature achieved locally, which complicates a fine control of therapeutic parameters *in situ*. Ximendes et al. combined in a recent work MNPs with infrared nanothermometers of Ag_2_S NPs that provided an efficient solution to this problem by monitoring the subcutaneous temperatures in real time, to build 2D thermal maps, which were used to accurately assess the therapeutic effect of the MNPs (Ximendes et al., 2021). Alternatively, the temperature at the surface of AMF-activated MNPs was also obtained with fluorescence probes by J. Ovejero et al. (Ovejero et al., 2021b). Moreover, to guarantee an efficient thermal treatment in tumors in a safe window of applicability in the clinical practice, a modelling of the heat distribution in tissues (*in silico* studies) is crucial. Rubia-Rodriguez et al. have explored collateral heating effects on prostheses that can affect the safety and efficiency of magnetic hyperthermia treatments of localized tumors (Rubia-Rodriguez et al., 2021b).

Luengo et al. enhanced the antibacterial properties of silver NPs in combination with iron oxide and its magnetic hyperthermia properties. The authors showed how the introduction of silver in the iron oxide particles had bactericidal activity against *Staphylococcus aureus* and *Escherichia coli*, and in addition, how the external magnetic fields enhanced this activity, demonstrating the synergistic properties of both materials when used in the same composite (Luengo et al., 2020).

In a recent study, MNPs were coated by a sonochemical method with a mesoporous silica surface in which the drug, DOX, could be loaded. The release of the drug used was dependent on pH, which showed effectiveness at acidic pH, proving the ultrasound synthesis as successful (Fuentes-García et al., 2021). In another strategy for DOX delivery, Lazaro-Carrillo et al. engineered a release mechanism based on iron oxide MNPs controlled by pH. The reductive environment of the cell was critical to diminish the side effects of the chemotherapy, increasing the effect against cancer cells (Lazaro-Carrillo et al., 2020).

Trabulo et al. developed a nanoformulation of MNPs with gemcitabine (chemotherapeutic drug) and anti-CD47 (adjuvant). The anti-CD47 antibody formulation showed efficient induction of apoptosis in cancer cells compared to free antibodies. In addition, the NPs were covered with BSA and polyethylene glycol (PEG) avoiding their rapid clearance and leading to a better efficacy (Trabulo et al., 2017).

MNPs can also be employed as smart delivery system for miRNAs. Lafuente-Gómez et al. developed maghemite core NPs loaded with immunomodulatory miRNA that induced a pro-inflammatory response in macrophages due to their load and specific coating (Lafuente-Gomez et al., 2022).

A promising strategy against cancer delivered by NPs is heterogeneous catalysis, which aims to target key chemical species of the tumor and generate *in situ* harmful biomolecules. Bonet-Aleta et al. engineered copper-iron oxide spinel NPs that effectively reduced glutathione levels and increased ROS and apoptotic pathways in cancer cells (Bonet-Aleta et al., 2022a). Furthermore, they investigated in depth the selective homo- and heterogenous catalytic processes undergoing in the tumor microenvironment, in which the higher glutathione levels are the main driving factor (Bonet-Aleta et al., 2022b). Glutathione is in a much higher concentration inside the cells than on the outside and it is largely responsible of the redox environment of the intracellular medium. This has also been exploited using iron oxide-MnO_2_ core-satellite shell NPs that undergo a chemical dissolution of the manganese dioxide shell when they are internalized by cells (Garcia-Soriano et al., 2022). This stimuli-responsive behavior changes the MRI contrast mode of the NPs and, at the same time, the iron oxide cores preserve their ability to kill cells through magnetic hyperthermia.

Another interesting property of MNPs is their antiviral activity, de Diego et al. showed that iron oxide NPs impair SARS-CoV-2 infection, highlighting their repurposing value as prophylactic agents against this viral infection (DeDiego et al., 2022).

Finally, the ultimate goal of research in nanomedical development is to reach clinical trials and improve current therapies. In this regard, it is worth highlighting the work done within the European project NoCanTher, where several Spanish institutions were involved. The consortium has been able to test the magnetic hyperthermia approach at Vall d’Hebron hospital (Barcelona) for the treatment of locally advanced pancreatic cancer. These types of studies are essential to make nanomaterial-based treatments a reality in the near future (Nanoscience, 2020).

### 2.3 Gold nanoparticles

Gold NPs (AuNPs) are especially relevant due to their ease of preparation, surface reactivity and unique optical properties (Garcia, 2011), (Goesmann and Feldmann, 2010; Wolfram and Ferrari, 2019). The small size of AuNPs, their biocompatibility, low toxicity and the possibility of simultaneous assembly of different molecular functionalities are attractive for biomedical use in therapy and sensing (Giner-Casares et al., 2016), (Saha et al., 2012), (Fabrizio et al., 2016), (Amendola et al., 2017), (Sperling et al., 2008), (Soenen et al., 2015). They are excellent candidates for PTT, biological imaging and optical sensing applications based on the localized surface plasmon resonance (LSPR) phenomenon, in terms of intrinsic properties as well as loading of different molecules, and they can also serve as contrast agents in computed tomography. Here we give an overview of the field given some examples of different AuNPs types but focusing on spherical colloids (Table 3).

**TABLE 3:** Summary of AuNPs with their therapeutic area and functionalization strategy.

Purpose	Cargo	Therapeutic area	Functionalization strategy	Ref.
Improve NP’s properties	Increase the cellular attachment	-	Gelation with polyoxometalates for encapsulation of Gold Nanorods into mucoadhesive hydrogel, allowing the attachment to the cytoplasmic membranes	Artiga et al. (2018)
	Enhance Adenovirus cellular uptake, distribution and therapeutic effect via surface modification with NPs	Cancer	Adenovirus decoration with PEGylated AuNPs carrying quaternary ammonium groups and RGD-motifs	Gonzalez-Pastor et al. (2021)
	To gain control over protein corona formation	-	Zwitterionic ligands based on oligocationic cages and negatively charged pyranine	Mosquera et al. (2020)
	Study physicochemical changes by tracking the spectral signatures using Hyperspectral-enhanced dark field microscopy	-	Polymer-coated gold/copper sulfide NPs	Zamora-Perez et al. (2021)
Enhance biological processes	Enhance immune response	HIV infection	AuNPs dually conjugated with IgG anti-HIVgp120 and IgG anti-human CD16, bringing together virus and NK cells to reinforce the immune response against virus	Astorga-Gamaza et al. (2021)
	Activation and expansion of T cells against tumors	Cancer	Nanostructured surfaces functionalized with the stimulating anti-CD3 antibody and the RGD peptide, plus costimulatory agents	Guasch et al. (2018)
Control hyperthermia	Monitor light-to-heat conversion of gold nanorods	-	Encapsulation of gold nanorods together with Nd-doped fluorescent NPs in a PLGA polymer	Rocha et al. (2016)
	Control local heating and nanothermometry	Brain cancer	Gold Nanostars (photothermal) combined with CaF_2_:Nd^3+,^Y^3^ luminescence NPs (thermometer)	Quintanilla et al. (2019)
	Nanothermometry Method	-	Gold nanorods and gold-iron oxide magnetic nanostars	Espinosa et al. (2021)
	Intercellular trafficking of gold nanostars as photothermal agents in cancer therapy		Gold nanostars functionalized with PEG-SH	Ahijado-Guzman et al. (2020)
Sustainable synthesis	Green synthesis of NPs for different purposes	Antimicrobial	Au and Ag NPs stabilized with “safely-obtained” biosurfactants	Gomez-Grana et al. (2017)
		Antiproliferative and Immunostimulative	Synthesis using an extract of *Saccorhiza polyschides* conferring a protective NP environment	Gonzalez-Ballesteros et al. (2021a)
		Cancer	Green synthesis of AuNPs with carrageenan from seaweed	Gonzalez-Ballesteros et al. (2021b)
Hyperthermia-triggered gene expression	Generation of cellular scaffolds and controlled gene expression	Cell therapy	AuNPs coated with poly-L-lysine through COOH-PEG-SH as covalent linker and thrombin to generate photothermal matrices	Escudero-Duch et al. (2019)

Purpose	Cargo	Therapeutic area	Functionalization strategy	Ref.
Drug delivery	Horseradish peroxidase		Covalent functionalization with horseradish peroxidase which oxidizes the prodrug indole-3-acetic acid (IAA) to release toxic oxidative species	Vivo-Llorca et al. (2022)
	Gemcitabine/gapmers		Combination of PEG, PEI and oligonucleotides electrostatically bound and released in reducing ambient	Garcia-Garrido et al. (2021)
	DOX		DOX into Gold Nanorods loaded-HSA NPs prepared through desolvation	Encinas-Basurto et al. (2018a)
	Docetaxel and DOX	Cancer	HSA/Chitosan NPs to encapsulate docetaxel and DOX-gold nanorods for chemotherapy and photothermal therapy	Villar-Alvarez et al. (2019)
	Antivascular drugs		Gold nanorods coated with mesoporous silica functionalized with aminopropyl groups and coupled to ICG photosensitizer. PEGylation with NHS-PEG-RGD	Paris et al. (2020)
	DOX		Nanorods coated with PSS DOX/PLL/human serum albumin layer-by-layer for chemotherapy and photothermal therapy	Villar-Alvarez et al. (2018)
	DOX		Fibrin hydrogel imbibing AuNPs and thermosensitive liposomes with DOX	Martin-Saavedra et al. (2017)
	DOX and SN38		Albumin-stabilized gold nanoclusters modified with drugs	Latorre et al. (2019)
Drug delivery	miRNA and SN38	Cancer	Conjugation of therapeutic oligonucleotides and SN38 using thiol moieties on AuNPs	Milan Rois et al. (2018)
	NK-Extracellular vesicles miRNAs		AuNPs functionalized with miRNAs via thiol modifications	Dosil et al. (2022)
	Calcein	-	Listeriolysin conjugated to the surface of AuNPs by functionalization with nitrile acetic acid	Plaza-Ga et al. (2019)
	Amikacin	Microbicidal	Gold nanostars functionalized with mercapto-poly(ethylene glycol)amino by ligand exchange and loaded with amikacin	Aguilera-Correa et al. (2022b)
	siRNA	Viral infection	AuNPs with cationic carbosilane dendrone coating	Pena-Gonzalez et al. (2017)
Smart delivery	Plasmid DNA	Gene therapy	Plasmonic PEGylated gold nanostars and gemini cationic lipoplexes	Sanchez-Arribas et al. (2021)
	Proteins	Biological therapy	PEGylated gold nanorods functionalized with a fluorescently labelled BSA cell penetrating peptide, and mixed with therapeutic proteins	Garcia et al. (2021)
	Antibodies	Cell-based therapies	Cell-derived NPs containing PEGylated gold nanorods for intracellular delivery of antibodies	Soprano et al. (2020)
	DOX	Cancer	Janus gold nanostars-mesoporous silica NP functionalized with a thiolated photolabile molecule	Hernandez Montoto et al. (2019)
	DOX in combination with PTT		Gold nanorods coated with silica and a thermosensitive polymer for drug delivery on demand upon irradiation	Villaverde et al. (2018)
	Bisbenzimide molecules		Thermoresponsive gold nanostars coated with ZIF-8 in combination with an amphiphilic polymer	Carrillo-Carrion et al. (2019)

The group of Liz-Marzan is a recognized reference in the synthesis of gold-based nanomaterials for multiple biomedical applications, including sensing, photothermia, and preparation of 3D scaffolds (Garcia-Lojo et al., 2019). For instance, they systematically investigated the synthesis of gold-branched nanostructures, such as nanostars with interesting optical properties related to LSPR and surface-enhanced spectroscopies, as excellent candidates for biomedical purposes (Barbosa et al., 2010). They are considered state-of-the-art NPs to be used as efficient agents for photothermal treatment at the NIR range employed as a single modality or combination with other therapeutic functionalities (Quintanilla et al., 2019), (Espinosa et al., 2016), (Villaverde et al., 2018).

**FIGURE 4:**
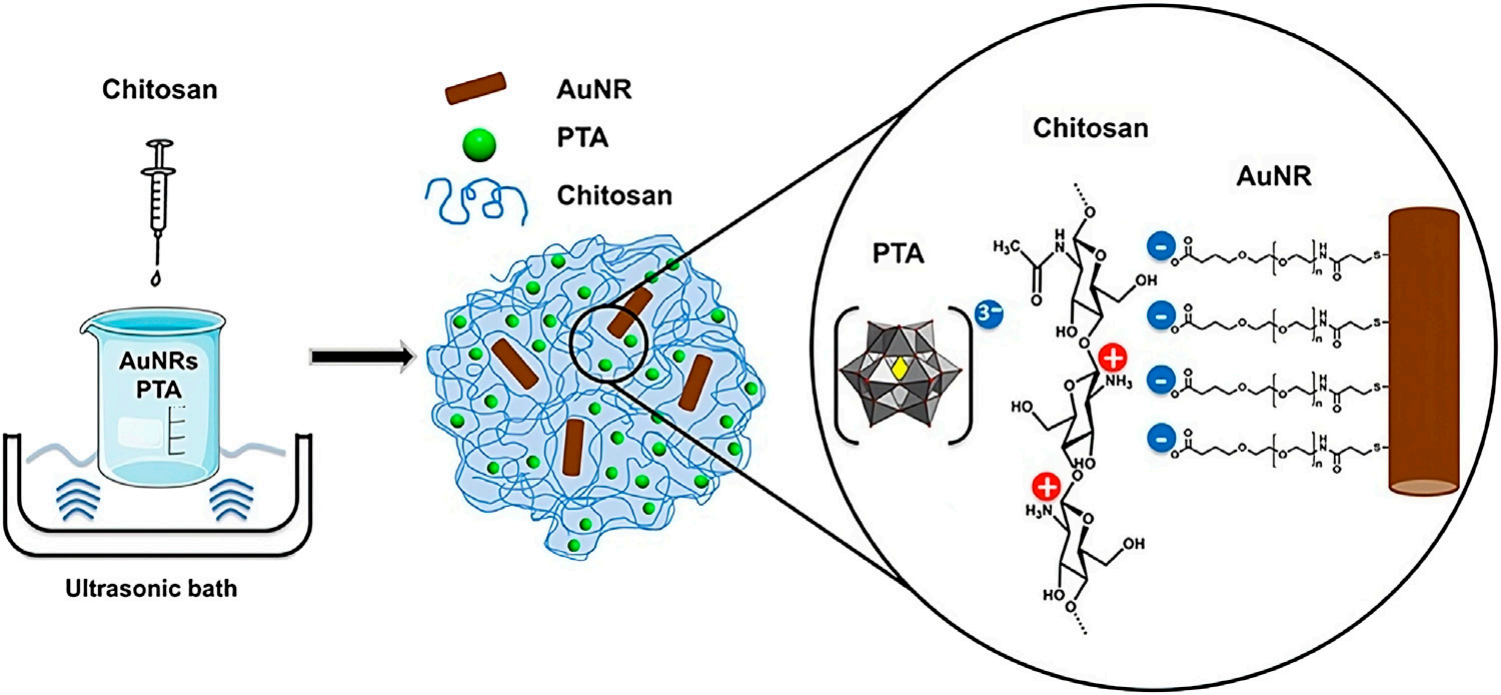
Synthesis of AuNR@CS hydrogel. Reprinted from Artiga et al. (2018)and licensed under the Creative Commons Attribution.

In order to enhance the cellular adherence of AuNPs, some strategies have been explored. For instance Artiga et al. encapsulated AuNPs inside a mucoadhesive chitosan hydrogel using polyoxometalates and phosphotungstic acid, showing that these containers can adhere to the cytoplasmic membrane of cells, enabling the thermoablating effect of the AuNPs without the need of cellular internalization (Artiga et al., 2018) (Figure 4). Gonzalez-Pastor et al. explored an interesting modification of Adenoviral vectors (Ad) for improving their uptake in resistant cells and their biodistribution. The authors proposed a strategy based on the modification of the Ad surface with 14 nm PEGylated AuNPs with quaternary ammonium groups and arginine-glycine-aspartic acid peptide motifs (or RGD-motif (Alipour et al., 2020)) to promote the attachment to cells via alternative cellular surface receptors, helped by the increase in positive charges. Modified vectors were tested in cellular models and in mice demonstrating their biocompatibility, high transduction efficiency, and antitumor activity (Gonzalez-Pastor et al., 2021).

As highlighted in MNPs, the shape of AuNPs also affects their biological behavior and therapeutic properties, and therefore a controlled synthesis with optimized purification methodologies is critical to obtain homogeneous NPs. Ramírez-Martínez et al. developed an efficient method to synthesize gold nanoprisms that showed reduced non-specific interactions with cells (Ramirez-Jimenez et al., 2020).

Enzyme prodrug therapy consists of selectively delivering an enzyme that activates a nontoxic prodrug into an active agent. Vivo-Llorca et al. functionalized the NPs with horseradish peroxidase, able to oxidize indole-3-acetic acid into toxic agents, and showed that this strategy presented high activity in 3D tumor models in which the three components on their own exhibit no therapeutic action (Vivo-Llorca et al., 2022).

Garcia-Garrido et al. studied drug delivery systems based on gold NPs tailored with low molecular weight polymers branched polyethylenimine and PEG. The system was able to deliver Gapmers targeting p53, reducing the chemoresistance to gemcitabine in mutant p53 cancer cells (Garcia-Garrido et al., 2021).

As is the case with every NP, the protein corona formed around NPs in biological media, modulates several key properties, including cellular internalization or clearance. Therefore, it is essential to study its formation, stability, and composition to understand NPs dynamics in cell and animal experiments. In this regard, Barbero et al. have studied the impact of common cell culture media elements in the formation of protein corona, and the mechanisms behind cellular penetration (Barbero et al., 2019), (Barbero et al., 2017), (Barbero et al., 2022). In this same subject, Mosquera et al. developed a strategy to control protein corona formation. The authors used AuNPs covered by an anionic dye (pyranine), which disrupts protein binding when a positively charged macromolecular cage is present. Zwitterionic surface ligands containing positive and negative charges will favor the formation of a protective hydration layer around the NPs. The authors demonstrate the reversibility of the system, which allows the control of corona formation through external additives. Applying this strategy the authors also increased (30-fold) the cellular uptake due to a synergistic effect between corona suppression and the charge switch from negative to positive at the NP surface. Finally, they explored its use in PTT, exploiting the conditional and enhanced cellular uptake of the system, this time using gold nanorods, with promising results (Mosquera et al., 2020).

Given the advantages of hybrid NPs, Encinas-Basurto et al. used DOX with gold NPs for PTT using human serum albumin NPs. When HeLa cells were treated with HSA-AuNR-DOX NPs, the cell viability was lower than the nanoplatform without DOX decreasing even further when the cells were irradiated (Encinas-Basurto et al., 2018a). Villar-Alvarez et al. also developed an hybrid nanocarrier based on human serum albumin/chitosan NPs that encapsulated free docetaxel and DOX-modified gold nanorods (DOX-GNRs) aiming to combine the chemotherapeutic properties of docetaxel and DOX with the plasmonic optical properties of GNRs for plasmonic-based PTT. This nanoformulation produced high cytotoxicity in breast cancer cells, and PTT enhances the cytostatic efficacy, with apoptosis being the main activated pathway (Villar-Alvarez et al., 2019). In a similar approach, Paris et al. engineered gold nanorods coated with a mesoporous silica shell to deliver two antivascular drugs with different mechanisms of action. The NPs also released heat and ROS through photothermal and PDT upon NIR light irradiation yielding remarkable results in a chicken embryo xenograft model (Paris et al., 2020).

In order to combine therapeutic plasmonic hyperthermia and DOX chemoaction, nanotransporters consisting of gold nanorods coated with poly(sodium-4-styrenesulfonate) (PSS)/DOX/hydrolyzed polylysine (PLL)/hyaluronic acid and (PSS/DOX/PLL)2/hyaluronic acid were developed by Villar-Alvarez et al. Hyaluronic acid targets CD44 receptors, which are overexpressed in some cancers. PTT induced cell necrosis, and apoptosis was promoted by DOX, resulting in a significant synergistic effect provided by a nano-based platform of targeted and multimodal controlled delivery (Villar-Alvarez et al., 2018). Astorga-Gamaza et al. also developed the synthesis of multivalent bispecific AuNPs to enhance the immune response towards HIV-expressing cells. They developed a cooperative adsorption methodology that allows the production of NPs with a 50:50 conjugation with two different antibodies that recognize the HIV gp120 protein and the CD16 receptor of natural killer cells. They performed a thorough characterization of the particles, which were able to promote specific cell-to-cell contact and induce a potent cytotoxic response (Astorga-Gamaza et al., 2021).

Martin-Saavedra et al. designed an hydrogel to incorporate AuNPs and thermosensitivie liposomes loaded with DOX. Upon NIR irradiation, the temperature rose locally releasing active DOX, whose delivery was dependent on the hydrogel composition and irradiation characteristics. Finally, the authors refined the system by incorporating copper sulfide NPs to create an easily biodegradable composite (Martin-Saavedra et al., 2017).

With the aim of controlling and monitoring the temperature of NPs reached during photothermal procedures in the tumor environment and, therefore, minimizing collateral effects during thermal treatments, plasmonic-mediated intracellular hyperthermia generated by Au nanomaterials has been tracked by nanothermometry methods. For instance, Rocha et al. have used Nd-doped Infrared-emitting NPs to monitor the light-to-heat conversion of Au nanorods during PTT (Rocha et al., 2016). Quintanilla et al. designed a hybrid probe for simultaneous plasmonic heating and NIR nanothermometry in glioma cells (Quintanilla et al., 2019). Finally, X-rays were also used to probe the local temperature of photoexcitated Au-based nanomaterials under NIR light for PTT, revealing significant nanothermal gradients (Espinosa et al., 2021).

Guasch et al., also investigated adoptive T cell therapy as a treatment for cancer, in an attempt to overcome the challenge of activating and expanding primary human T cells *in vitro*. They performed a method for activating primary human CD4^+^ T cells *in vitro* functionalizing nanostructured surfaces. These surfaces consist of covalently functionalized RGD on rigid TiO_2_ surfaces decorated with arrays of AuNPs cell-linked to the stimulating antibody anti-CD3. They demonstrated that the combination of prestimulatory steps, nanostructure surfaces, and costimulatory compounds has an effect on the activation and proliferation of cells (Guasch et al., 2018).

Multidrug resistance is one of the problems of chemotherapy that reduces the efficacy of treatment, and nanocarriers can be used to enhance permeability and retention effect at the target site. Latorre et al. selected two chemotherapeutic drugs, DOX and the camptothecin analogue (CPT) SN38, for the functionalization of albumin-stabilized gold nanoclusters (AuNCs) using tailored linkers. The drugs were released when exposed to different stimuli, such as glutathione and acid pH, leading to a potent antitumor activity. Furthermore, this system showed antineoplastic activity against cancer stem cells (Latorre et al., 2019).

Recently, there has been wide interest in the nanomaterials community to synthesize NPs in a sustainable manner, reducing the use of toxic chemicals and solvents. In this regard, Gomez-Graña et al. explored the possibility of synthesizing gold and silver NPs using a lipopeptide biosurfactant extracted from corn steep liquor. The silver NPs showed antimicrobial properties against *Escherichia coli* that was greater than similar citrate-stabilized NPs, enhancing the application of sustainable methodologies in NP synthesis (Gomez-Grana et al., 2017). Gonzalez-Ballesteros et al. also synthesized gold and silver NPs in bionanofactories, aiming at developing environmentally friendly processes for NPs synthesis. They characterized the NPs obtained and demonstrated that the particles showed antiproliferative properties and could also serve as immunostimulant agents (Gonzalez-Ballesteros et al., 2021a). In another related work, Gonzalez-Ballesteros and colleagues performed a green synthesis of AuNPs that were decorated with carrageenan extracted from red seaweed. The NPs showed relevant antioxidant and antitumoral properties, highlighting the beneficial effect of NP loading of the active compound (Gonzalez-Ballesteros et al., 2021b).

Regarding delivery, AuNPs have demonstrated to be effective carriers for a wide variety of oligonucleotides. For example, siRNAs are interesting molecules capable of modulating gene expression. One of the critical factors for this strategy to be effective is choosing an optimal strategy to link the siRNA to the AuNPs, and several chemistries have been developed to form stable complexes with proven activity in cellular and animal models (Tortiglione and de la Fuente, 2019). Sánchez-Arribas et al. developed a strategy combining plasmonic gold nanostars and gemini cationic lipoplexes to release plasmid DNA upon irradiation (Sanchez-Arribas et al., 2021). Interestingly, this strategy exploits the release of a DNA plasmid “on demand” upon external stimuli.

Similarly, Milan-Rois et al., developed a strategy for the delivery of four miRNA downregulated in uveal melanoma, and other cancers, in combination with SN38, a topoisomerase I inhibitor. The study showed a synergistic effect between the four oligonucleotides and chemotherapeutic drug conjugated to the AuNPs (Milan Rois et al., 2018). In the same pathology, Ahijado-Guzman et al. explored the potential of gold nanostars as efficient plasmonic PTT using a non-harmful laser irradiation (Ahijado-Guzman et al., 2020). Their results show how these cancer cells can release and uptake the NPs achieving effective PTT even in non-preloaded cells.

Dosil et al. have demonstrated how AuNP-based delivery of specific NK-extracellular vesicles-miRNAs regulates immune responses related to Th1 and recapitulated this phenomenon in animal models. Th1 cells directly killed tumor cells via the release of cytokines that activate death receptors on the tumor cell surface, representing a potential immunomodulatory strategy against diseases (Dosil et al., 2022).

Endosomal escape of the transported cargo is another important feature of NP delivery, to guarantee active functionality in the cytosol. In a recent work, Plaza Ga et al. (Plaza-Ga et al., 2019), explored a mechanism used by the bacterial pathogen *Listeria monocytogenes* through a toxin called Listeriolysin O. They conjugated this protein to the surface of AuNPs and observed how upon endosomal acidification, the protein disassembles from the NPs to form a pore in the endosomal lipid bilayer enabling the escape of NPs. Within the same focus, and to improve the delivery of proteins, Garcia et al. developed gold nanorods modified with a cell-penetrating peptide that, upon NIR irradiation in the safe second biological window, releases the protein cargo in a controlled spatial and temporal manner (Garcia et al., 2021).

Smart delivery or delivery after external stimuli offers exciting methodologies for delivering cargo into the cytosol on demand. Soprano et al. developed cell-derived NPs that contained gold nanorods in their structure that enabled the release of non-permeant antibodies into the cytosol of cells. The nanocarriers were responsive to NIR irradiation, which proved safe for cells at the conditions needed for cytosolic delivery (Soprano et al., 2020).

Escudero-Duch and collaborators developed a NIR responsive hydrogel based on fibrin, and hollow poly-L-lysine covered gold NPs. The *in-situ* polymerization of fibrin upon NIR irradiation yields a hydrogel potentially suitable for its use as a scaffold in regenerative medicine. This hydrogel tested in cellular and animal models showed good biocompatibility and allowed the spatial patterning of transgene expression triggered by heat (Escudero-Duch et al., 2019).

Hernández-Montoto et al. prepared Janus gold nanostars MSNs loaded with DOX, and equipped with a cyclodextrin supramolecular gatekeeper. NIR light triggered the release of succinic acid that enabled gate opening and cargo delivery. This strategy enables to use AuNPs as photochemical transducers able to release a chemical messenger upon NIR irradiation. The Janus NPs showed a reduction in cell viability, proving their potential as smart delivery materials (Hernandez Montoto et al., 2019).

In an attempt to improve the available treatments for melanoma, which is highly resistant to cytotoxic agents after metastasis, Villaverde et al. engineered gold nanorods coated with silica and a thermosensitive polymer conjugated to NAPamide, a selective targeting agent for alpha melanocytes. Thus, these NPs exerted a synergistic effect of the cytotoxic DOX and PTT (Villaverde et al., 2018).

Carrillo-Carrión et al. investigated the combination of gold nanostars with metal organic frameworks based on zeolitic imidazole and an amphiphilic polymer to afford thermoresponsive nanocomposites. They demonstrated their stability and the release of cargo inside cells (Carrillo-Carrion et al., 2019).

Zamora-Pérez et al. employed Hyperspectral-Enhanced Dark Field Microscopy (HEDFM) to test the dynamics of Au/CuS NPs directly, based on the changes in the scattering of the nanomaterial in different physiological conditions. Changes in the scattering profiles of NPs could be used as indicators of their performance as photothermal probes. The authors demonstrated how the combination of plasmonic NPs with HEDFM informs of the behavior of intracellular NPs to optimize their functionality for nanomedical applications (Zamora-Perez et al., 2021).

Aguilera-Correa et al. also developed gold nanostars and tested their antimicrobial properties alone and loaded with a potent and widely used antibiotic. The gold nanostars *per se* did not show antimicrobial activity, however in combination with amikacin inhibited the growth of bacterial biofilm of carbapenem-resistant *Klebsiella pneumoniae* strain, suggesting that the NPs facilitate the entrance of the therapeutic agent into the biofilm (Aguilera-Correa et al., 2022b).

Finally, Peña-Gonzalez et al. engineered AuNPs and AgNPs with a cationic carbosilane dendrone coating that improved their delivering capabilities, and characterized their interactions with erythrocytes, platelets, and peripheral blood mononuclear cells. The NPs showed to have a safe profile in these systems and proved successful in cell delivery of siRNA against HIV (Pena-Gonzalez et al., 2017).

### 2.4 Silver nanoparticles

Similar to gold NPs, silver NPs (AgNPs) have unique and useful properties and are being used in several consumer products such as textiles or home appliances (Ahamed et al., 2010). The antibacterial properties of silver have been known for centuries (McGillicuddy et al., 2017), and colloidal silver has been used by humans for more than 150 years for the treatment of wounds and infections (Reidy et al., 2013). Interestingly, this biological activity is gaining significant interest among researchers due to the resistance developed by different pathogens to current antibiotics. In this regard, some developments carried out by Spanish scientists are mentioned below (Table 4).

**TABLE 4:** Summary of AgNPs with their therapeutic area and functionalization strategy.

NP Type	Purpose	Therapeutic area	Therapeutic agent	Functionalization strategy	Ref
AgNPs	Anti-amoebic contact lens solution	Keratitis	-	Commercially available AgNPs	Hendiger et al. (2021), (Hendiger et al. (2020)
	Anti-amoebic contact lens solution	Keratitis, encephalitis	Tannic acid	Tannic acid-modified AgNPs	Padzik et al. (2018)
	Prevention of implant-associated infection	Antibacterial	-	AgNPs were synthesized by laser ablation in de-ionized water	Perez-Tanoira et al. (2022)
	Low cost antitumor tool	Cancer	Acetogenin rich extracts	Extracts from leaves and peel of *A. muricata* were used to synthesize AgNPs	Gonzalez-Pedroza et al. (2021)
	Anti-biofilm treatments in chronic wound infections	Antibacterial	Enzymes (α-amylase, cellulose, DNase I and proteinase K)	Enzyme-coated AgNPs	Rubio-Canalejas et al. (2022)
	Combination of two antibacterial agents (probiotics and AgNPs)	Antibacterial (topical)	Probiotics	Probiotics and AgNPs in a matrix as bacterial cellulose	Sabio et al. (2021)
GSH-Ag NPs	Improve efficacy for multidrug resistant bacteria	Antibacterial	-	Glutathione for stabilization	Silvan et al. (2018)
AgNP@nanoMOF	Combating bacterial biofilms		-	Silver impregnated nanoMOF thin film functionalized with DNase I	Arenas-Vivo et al. (2019)
Silver nanorings	Study a novel type of AgNPs as an antimicrobial therapy	Antifungal, antiamoebic	-	Silver nanorings which have a filament diameter of 80 nm and a ring diameter of between 12 and 18 µm	Gonzalez-Fernandez et al. (2022)
PLGA@Ag_2_S and PLGA@Ag_2_S@SPION	Hybrid system for chemotherapy	Cancer	Maslinic acid	Combination of Ag_2_S NPs and SPIONs by electrospraying into a PLGA matrix loaded with maslinic acid (PLGA@Ag2S@maslinic acid)	Alvear-Jimenez et al. (2022)
AgNPs and AuNPs	Nanoparticles as delivery systems for cancer cells	Cancer	Raltitrexed	AuNPs and AuNPs functionalized with raltitrexed	Morey et al. (2021)
AgNPs nanofluid	Photothermal agent acting as nanothermometer		-	AgNPs were functionalized with HS-PEG-OMe	Mendez-Gonzalez et al. (2022)

Silvan et al. designed AgNPs stabilized with glutathione and evaluated their efficacy against multidrug-resistant *Campylobacter* strains that were extracted from chicken samples. While the NPs were able to inhibit bacterial growth, the mean minimal inhibitory concentration resulted cytotoxic for three different human intestinal cell lines tested. This effect highlights the importance of further safety experiments to assess the practical potential of AgNPs therapeutic effects (Silvan et al., 2018).

Sabio et al. designed a two-sided material combining AgNPs on one side and living probiotics on the other, with antibacterial capacity against *Pseudomonas aeruginosa* (Sabio et al., 2021). AgNPs have also been explored for their anti-amoebotic properties against pathogens responsible for keratitis. In this regard, Hendiger et al. evaluated the activity, cytotoxicity, and anti-adhesive properties of AgNPs included in contact lens solutions against the *Acanthamoeba castellanii Neff* strain (Hendiger et al., 2021), (Hendiger et al., 2020). The presence of the AgNPs showed a significant increase in anti-amoebic activity, without increasing the overall cytotoxicity, decreasing the risk of *Acanthamoeba keratitis* infection. Padzik et al. also employed AgNPs conjugated with tannic acid as potential agents against *Acanthamoeba spp* (Padzik et al., 2018).

In the case of peri-implantitis due to biofilm deposits, Pérez-Tanoira et al. immobilized AgNPs in titanium, demonstrating its beneficial effect in reducing the biofilms stablished by *S. aureus* and by mixed oral bacterial flora (Perez-Tanoira et al., 2022). Other anti-biofilm strategies involve the use of silver-containing nanoscaled Metal Organic Frameworks (MOFs) against *S. aureus* biofilm. Arenas-Vivo et al. demonstrated the use of AgNPs functionalized with DNase I decreasing the *S. aureus* biofilm viability more than using the antibiotics alone (Arenas-Vivo et al., 2019). Rubio-Canalejas et al. pointed out that clinical treatment combining antibiofilm enzymes and antibiotics may be essential to eliminating chronic wound infections (Rubio-Canalejas et al., 2022). Finally, González-Fernández et al. demonstrated how silver nanorings are capable of totally inhibiting the germination of *A. castellanii* cysts (Gonzalez-Fernandez et al., 2022).

In regards to drug delivery, the formation of hybrid Ag_2_S NPs with poly(lactic-co-glycolic acid) (PLGA) by electrospray allows for the encapsulation of drugs such as maslinic acid (MA). The anticancer drug showed an efficient encapsulation and controlled release in cellular models (Alvear-Jimenez et al., 2022).

Morey et al., modified silver and gold NPs using cysteine to bind raltitrexed to the surface of NPs and tested them in A549 and HTC-116 cells lines. Silver raltitrexed NPs inhibited cancer cell viability (Morey et al., 2021). Other modifications, such as the functionalization of AgNPs with *Annona muricata* plant antitumoral extracts, have been prepared and tested by González-Pedroza et al. as promising antitumoral nanoformulations (Gonzalez-Pedroza et al., 2021).

Finally, AgNPs can be used as luminescent biofluids capable of acting as photothermal agents and nanothermometers. Mendez-Gonzalez et al. showed how a nanofluid containing AgNPs had improved properties compared to a combination with magnetic nanoflowers and showcases their use for hyperthermia in brain tumors (Mendez-Gonzalez et al., 2022).

## 3 Organic nanoparticles

Organic NPs have synthetic or natural organic components such as carbohydrates, proteins, peptides, or lipids (Romero et al., 2012). Their biodegradable composition, together with the relatively simple encapsulation of drugs, make them the preferred drug delivery systems. The current advances in the use of protein, polysaccharide, polymeric and lipid NPs carried out in Spanish institutions are discussed below, and summarized in tables at the end of each section.

### 3.1 Protein nanoparticles

Protein NPs are of great interest in nanomedicine due to the intrinsic properties of proteins, such as their biocompatibility and biodegradability. Proteins possess different functional groups located in the side chains of amino acids that can be exploited for chemical conjugation. They also contain hydrophilic and hydrophobic regions that can be used to interact with hydrosoluble or insoluble compounds. Finally, they have tunable structures that can be obtained by protein engineering, expanding their applications (Table 5).

**TABLE 5:** Summary of protein NPs with their therapeutic area and synthesis strategy.

Protein	Purpose	Therapeutic area	Therapeutic Agent	Synthesis/Encapsulation strategy	Ref
Albumin	Drug delivery/smart release	Cancer	DOX	NPs stabilized by redox crosslinker to increase drug release in cancer cells	Prajapati et al. (2021)
	Increase efficacy and biodisponibility of AZD8055	Uveal melanoma	AZD8055	Gold nanoculsters stabilized by albumin. Cargo conjugation by disulfide bonds	Latorre et al. (2021)
	Increase selectivity of nanoparticle delivery against tumor	Cancer	PTX	Folate-targeted NPs based on BSA and alginate and stabilized by amide bonds using ethylenediamine	Martinez-Relimpio et al. (2021)
	Develop tumor-targeted BSA NPs		-	BSA NPs functionalized with chlorosydnone to click with anti-PD-L1 antibodies with dibenzocyclooctyne moeities	Gerke et al. (2022)
Protamine	Insulin oral delivery	Diabetes	Insulin	Nanocarriers with oily core and protamine/PSA shell	Thwala et al. (2018)
	Vaccine adjuvant	Influenza	Different antigens	Nanocarriers with oily core and protamine shell	Gonzalez-Aramundiz et al. (2017), Gonzalez-Aramundiz et al. (2018)
Polyarginine	Oral peptide delivery	Diabetes	Insulin and oleic acid	Nanocapsules with oily core and polyarginine shell	Niu et al. (2017)
Zein	Insulin delivery		Insulin	Zein NPs coated with PEG and synthetic polymers	Reboredo et al. (2021), Inchaurraga et al. (2020)
				Zein NPs coated with PEG and Gantrez AN-PEG	Martinez-Lopez et al. (2021)
Engineered peptides	Toxic proteins delivery	Cancer	Diphteria and *P.aeruginosa* toxins	Toxic peptides self-assembled in NPs with specific targeting regions	Sanchez-Garcia et al. (2018), Volta-Duran et al. (2021)
			29 amino acid-segment of the helix α5 from the human BAX protein		Sanchez-Garcia et al. (2020)
			Exotoxin A from *P.aeruginosa*		Falgas et al. (2020)
			Diphteria toxin		Pallares et al. (2021)
			Monometyl Auristatin E (MMAE)		Pallares et al. (2020)

**FIGURE 5:**
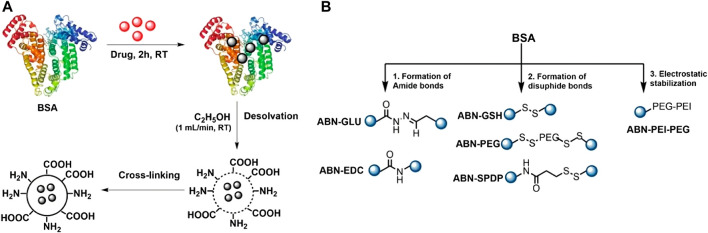
Albumin NPs synthesis. Reprinted from Prajapati et al. (2021) and licensed under the Creative Commons Attribution.

Several proteins have been used to create NPs for their use in nanomedicine, being albumin one of the most successful ones. It is naturally present in the blood, so it can avoid immunogenic reactions, increasing the circulation time of their cargoes. It is a natural vehicle especially suited for the interaction with hydrophobic molecules. Furthermore, human cells present albumin receptors, namely, gp60 and SPARC (Prajapati and Somoza, 2021). As these receptors are overexpressed in cancer cells, albumin NPs can be used to target tumor sites. This strategy has been successfully used in the formulation of Abraxane, which has been approved for the treatment of several tumors (Trabulo et al., 2017). Albumin is a monomeric protein unable to form NPs by itself, therefore several methods have been used to form albumin nanostructures, being desolvation the most common one. For example, Prajapati et al. used this method to encapsulate DOX, a drug limited by its toxicity. Its encapsulation inside of NPs enabled to target tumor cells specifically, increased the drug efficacy, and decreased its toxicity. Furthermore, they stabilized the NPs employing several cross-linkers, showing that a redox-dependent crosslinker (SPDP, N-succinimidyl 3-(2-pyridyldithio) propionate) increased drug release in cancer cells, due to their enhanced redox environment compared to non-cancer cells. Their nanoformulation showed toxicity in breast cancer cells and a negligible effect in non-tumoral cells, presenting a potential use for the treatment of breast cancer (Prajapati et al., 2021) (Figure 5). The same group also used albumin-based nanostructures for uveal melanoma treatment. In another article, they studied the use of albumin to deliver AZD8055, a potent inhibitor of the mTOR pathway that is overexpressed in the pathology and is critical in tumorgenesis. They produced gold nanoclusters stabilized by albumin, while the drug was conjugated externally using disulphide bonds. The lately thiol-dependent conjugation of the drug allowed its specific release in the cytoplasm of cancer cells. The authors showed that their nanostructures had anti-tumoral activity in mice models, using a dose 23-fold lower than previously reported (Latorre et al., 2021). The conjugation of folate to NPs based on BSA and alginate as an active targeting strategy for the delivery of PTX was showed by Martinez-Relimpio et al. which resulted in an increased uptake of the NPs by cancer cell lines, as there is an overexpression of folate receptors (Martinez-Relimpio et al., 2021). To exploit BSA NPs delivery possibilities Gerke et al. developed a simple methodology with clickable anti-PD-L1 antibodies, showcasing the versatility of this bioorthogonal design (Gerke et al., 2022).

Hydrophilic proteins with multiple arginine residues, such as protamine and polyarginine, are widely used to form NPs as their positive charge confers membrane-translocation properties (Thwala et al., 2018). Similarly to albumin, it does not form NPs by itself, so a solvent displacement method must be used to create protamine nanocapsules. NPs contain an oily core and a protamine coating. Thwala et al. used them for the delivery of insulin, a highly used protein that cannot be administered orally. The authors used a protamine/PSA shell that controls the release of insulin from the moment the NPs are administered orally until reaching the intestine (Thwala et al., 2018). Furthermore, the insulin transport across mucus layers can be increased by incorporating in the formulation penetration enhancers such as oleic acid (Niu et al., 2017). The same group used protamine nanocapsules loaded with antigens as an alternative to vaccine adjuvants. This system can load multiple antigens, be lyophilized, and trigger the immune response. These properties have been shown in particles loaded with influenza hemagglutinin antigen, and particles containing hepatitis B virus surface antigen (Gonzalez-Aramundiz et al., 2017; Gonzalez-Aramundiz et al., 2018). According to the results, the protamine nanocapsules showed the ability to enter macrophages without toxicity and produced an important immune response against influenza (Gonzalez-Aramundiz et al., 2017).

Zein is a small hydrophobic protein that, in contrast to albumin and protamine, can easily self-assemble, forming colloidal NPs in the aqueous phase. Zein NPs coated with PEG and Gantrez AN-thiamine have been used to deliver insulin. They have an enhanced permeation within the mucus and intestinal absorption, which decreases the glucose level in blood (Inchaurraga et al., 2020; Reboredo et al., 2021). In particular, the Gantrez conjugate reduced the accumulated fat in *Caenorhabditis elegans* (Martinez-Lopez et al., 2021). Given this promising results a double blind clinical trial has been designed by Clínica Universidad de Navarra to determine whether this particles are able to provide glycemic control in patients (NT: 05560412) (Clinica Universidad de Navarra UdN, 2022b).

Another interesting kind of proteins are the ones considered self-delivered nanoscale drugs that, at the same time, can self-assemble in NPs. With this in mind, Sanchez-García et al. fused bacterial toxin peptides to a N-terminal cationic T22 peptide and a C-terminal region with 6 histidines. These engineered peptides self-assemble in nanostructures by the interaction of the N- and C-terminal regions. T22 peptide can recognize CXCR4, a receptor overexpressed in cancer cells, providing specific tumor targeting, while the toxin peptide promotes general cell death (Sanchez-Garcia et al., 2018). The bacterial toxins can be exchanged for human pro-apoptotic peptides with similar results (Sanchez-Garcia et al., 2020). These nanostructures were successfully applied to several cancer models in mice, such as colon, (Sanchez-Garcia et al., 2018), lymphoma, (Falgas et al., 2020), and leukemia (Pallares et al., 2021). Volta-Duran et al. (Volta-Duran et al., 2021) designed a method to deliver anticancer drug pairs that consist of a tumor-targeted protein NP based on two microbial toxins, exotoxin A and diphtheria toxin, chemically coupled with oligo-floxuridine and monomethyl auristatin E respectively. These nanoformulations were able to internalize into target cells and had a biological impact. Unfortunately, the chemical conjugation annulled the activity of the toxins. Pallares et al. synthesized a nanoconjugate that contained GFP instead of a bacterial toxin, covalently labelled with auristatin, a potent antimitotic agent. With this treatment, they were able to significantly reduce and control myeloid leukemia dissemination (Pallares et al., 2020).

### 3.2 Polysaccharide nanoparticles

Polysaccharides are biomacromolecules formed by sugars found in every living organism. They are non-toxic or immunogenic and are a better biocompatible alternative to synthetic polymers. In addition, they are highly versatile, varying in molecular weight, branch degree, and functional groups, and can be tuned to deliver different cargo (Serrano-Sevilla et al., 2019; Shokrani et al., 2022). In Spain, many polysaccharides have been used to form nanostructures with biomedical applications, such as chitosan, hyaluronic acid or cellulose as reviewed here (Table 6).

**TABLE 6:** Summary of polysaccharide NPs with their therapeutic area and synthesis strategy.

Saccharide	Purpose	Therapeutic area	Therapeutic Agent	Synthesis/Encapsulation strategy	Ref.
Chitosan	Drug delivery	Wnt signaling	Alsterpaullone	Oily core chitosan NPs formed by nanoemulsion	Ambrosone et al. (2020)
	Drug delivery to brain	Neurodegenerative diseases	PH797804	Oily core chitosan NPs formed by nanoemulsion	Casadome-Perales et al. (2019)
	Delivery to vaginal mucosa	Microbicidal	Peptide	NPs formed by inotropic gelation of chitosan with TPP method	Marciello et al. (2017)
	Nanovaccine	HIV	HIV antigen	NPs formed by ionic complexation of positive chitosan with negative hyaluronic acid or dextran	Dacoba et al. (2019), Dacoba et al. (2020a)
Mannose dendrons	Tolerance for allergens	Immune response modulation	Prup3 allergen peptide	Prup3 peptide bound to a mannose dendron structure (D_1_ManPrup3 and D_4_ManPrup3)	Rodriguez et al. (2019)
Hyaluronic acid	Tumor penetration	Cancer	Docetaxel	Hyaluronic acid modified with maleimide and with peptide tLyp1 on the shell	Teijeiro-Valino et al. (2019)
	Drug delivery	Cancer	Docetaxel	Hyaluronic acid functionalized by hydrophobic side chains	Cadete et al. (2019)
	Intraocular Drug delivery	Ocular inserts	Ferulic acid and peptide ε-polylysine	Electrospunned Hyaluronic acid by using PVP as excipient	Grimaudo et al. (2020)
	Drug delivery	Inflammatory joint diseases	Anti-inflammatory drugs	Hydrogels formed by Hyaluronic acid and fibrin with Hyaluronic acid NPs	Storozhylova et al. (2020)
	Drug delivery at infection site	Antibacterial	Clarithromycin	Clarithromycin-loaded papain-modified ureido-conjugated thiolated hyaluronic acid-co-oleic acid (CLR-PAP-Ur-thHyaluronic acid-co-OA) nanomicelles	Qaiser et al. (2022)
Cellulose	Drug delivery	Cancer	DOX	CNC modified with APTES and functionalized with FA, followed by incorporating Carbon dots and DOX	Do et al. (2021)
Cyclodextrin	Delivery of DNA to spleen	Gene therapy	DNA	Cyclodextrin-based molecular NPs through covalent dimerization	Gallego-Yerga et al. (2018)
Inulin	Improve lymphatic accumulation	Nanovaccine development	-	Small and negatively charged inulin nanocapsules	Crecente-Campo et al. (2019)

Chitosan is a derivative of the natural polysaccharide chitin, which is the second most abundant polysaccharide in the world after cellulose. Chitosan has many interesting properties, including biocompatibility, biodegradability, antibacterial effect, and muco-adhesion, and it is widely used in food, cosmetics, and biomedical applications. Chitosan has a high concentration of reactive amino groups along its backbone, conferring a high positive charge that promotes its interaction with biological tissues (Frigaard et al., 2022). The excellent properties of chitosan NPs for intracellular delivery have been exploited by Ambrossone et al. They showed that oily core chitosan nanocapsules synthesized by nanoemulsion efficiently delivered alsterpaullone, a Wnt signaling agonist, into the model organism *Hydra vulgaris*. They also studied the characteristics of the intracellular delivery with Nile red-loaded NPs. Their methodology resulted in a more efficient manner of activating Wnt pathway than free alsterpaullone at the same concentration (Ambrosone et al., 2020). Similarly, Montero et al. developed chitosan-BSA NPs and studied their potential as vehicles with different combinations, demonstrating their potential for drug delivery (Montero et al., 2019).

Chitosan NPs also enable the transportation of agents into the brain, given the difficulty to target this organ. As a proof of concept, Casadomé-Perales et al. demonstrated the inhibition of p38 MAPK, an enzyme that is commonly dysregulated in several neurodegenerative diseases. They encapsulated PH797804, an inhibitor of this enzyme, in NPs with a nanoemulsion core. The intranasal delivery of the NPs enabled the inhibition in different parts of the brain in animal models, showing that it could be an efficient strategy for brain delivery (Casadome-Perales et al., 2019).

Marciello et al. used chitosan NPs to deliver a model peptide through vaginal mucosa, given the interest of microbicides delivery for the prevention and treatment of sexually transmitted diseases. The NPs contained chitosan and ascorbate, with insulin as the model cargo. Then, the NPs were incorporated in sponge-like cylinders made of mannitol, sucrose, and gelatin B to control their release in the vaginal environment (Marciello et al., 2017).

The fact that chitosan NPs can encapsulate peptides opens the possibility of their use as nanovaccines. As mentioned previously, using NPs as vaccines improves both the administration and the activation of the targeted immune cells, which results in greater efficacy. The role of chitosan in these formulations has shown to enhance the activation of the adaptive immune response (Moran et al., 2018).

The generation of an effective HIV vaccine is still an important health challenge, to which several nanotechnological approaches are being developed. According to previous works, the peptide sequences around the protease cleavage sites have been proposed as a target for HIV vaccines. Dacoba et al. developed different chitosan NPs loaded with the HIV peptide PCS5. The NPs were formed by ionic complexation, mixing the positive polysaccharide chitosan with negative ones like Hyaluronic acid or dextran. Furthermore, they tested if the presence of poly (I:C), an immunomodulatory molecule, had any effect. All the NPs were able to induce humoral responses against the antigen (Dacoba et al., 2019). However, they showed that the binding of the antigen, the presence of poly (I:C), and the nature of the polysaccharides influence the type of immune response, such as the kinetics of the effector T cell responses. The results suggest the possibility of developing a nanovaccine against HIV and its translation into clinical trials (Dacoba et al., 2020a). The same group showed that NPs made of chitosan and the anionic carboxymethyl-β-glucan, which accumulate in the lymph nodes, promoted the accumulation of the NPs in draining lymph nodes and exerted an immune response. The NPs were formed using the ionic complexation method, and loaded with ovalbumin (Frigaard et al., 2022).

Polysaccharide NPs loaded with antigens can also be used to induce tolerance for allergens. Rodriguez et al. explored the capabilities of several mannose nanostructures to serve as an efficient platform to generate specific recognition without the need for additional adjuvants. In this particular case the treatment developed prolonged protection against allergen exposure without any sign of anaphylaxis (Rodriguez et al., 2019).

Hyaluronic acid is an anionic polysaccharide, a glycosaminoglycan, consisting of disaccharide repeating units of β-1,4-D-glucuronic acid-β-1,3-N-acetyl-D-glucosamine. It has a high binding affinity towards the CD44 receptor, highly expressed in cancer cells (Liu and Huang, 2022). It can be used as shell in oil-based NPs, by the solvent displacement technique. Teijeiro-Valino et al. used this kind of NPs for the encapsulation of the anti-cancer drug docetaxel. Furthermore, they decorated the hyaluronic acid shell with the tumor homing peptide tLyp1. Their formulation increased penetration in the tumor and anti-cancer activity in lung and pancreatic cancer mice models (Teijeiro-Valino et al., 2019).

Cadete et al. used a modification of hyaluronic acid consisting of the addition of hydrophobic side chains, like dodecyl, to promote the self-assembly of NPs without the use of surfactants. This strategy decreased the cytotoxicity of the NPs, while showing an improved intracellular drug delivery (Cadete et al., 2019).

Delivery of drugs on the eye surface can be achieved by flexible electrospun nanofibers, which are able to adapt and persist on the eye surface whilst the drug is released. Grimaudo et al. overcame the incapability of hyaluronic acid to be electrospunned, by using PVP as an excipient. This resulted in creating hyaluronan nanofibers, capable of delivering the antioxidant ferulic acid and the antimicrobial peptide ε-polylysine at the same time (Grimaudo et al., 2020).

In another study, Storozhylova et al. engineered hydrogels formed by Hyaluronic acid and fibrin, with hyaluronic acid nanocapsules loaded with anti-inflammatory drugs. They were used to improve intra-articular administration, showing a rapid efflux of the administered drugs. This system could relieve the inflammatory conditions of large joints (Storozhylova et al., 2020).

Qaiser et al. developed three types of nanomicelles formulations to synthesize a targeted, mucoadhesive and mucopenetrating drug delivery system. The goal was to encapsulate clarithromycin, an antibacterial drug, to improve its residence time at the *Helicobacter pylori* infection site. They concluded that clarithromycin-loaded papain-modified ureido-conjugated thiolated hyaluronic acid-co-oleic acid (CLR-PAP-Ur-thHA-co-OA) nanomicelles could be used as nanocarriers for the treatment of *H. pylori* infection, due to their mucopenetration, mucoadhesion properties, stability, and extended drug release (Qaiser et al., 2022).

Cellulose is the world’s most abundant polysaccharide. It is a linear polymer composed of repeating units of two anhydroglucose rings. Its abundance and biocompatibility make nanocellulose a good candidate for biomedical applications (Nicu et al., 2021). Recently, Do et al. developed a modified cellulose nanocrystal (CNC) for the delivery of anti-cancer drugs. They engineered nanoplatforms based in modified CNCs with APTES to improve their dispersibility. These CNCs were covalently functionalized with folic acid (FA), followed by the incorporation of Carbon dots and the drug DOX, by electrostatic interaction. These CNCs may be promising nanoplatforms to be used both in chemotherapy and PTT against cancer (Do et al., 2021).

Gallego-Yerga et al. prepared DNA-cyclodextrin NPs to improve gene therapy. They controlled the morphology of the complexes to study how the shape affects the transfection properties. They found several complexes that exhibited highly efficient transgene expression and were able to deliver DNA to the spleen in a tissue-specific manner in animal models (Gallego-Yerga et al., 2018).

Finally, inulin is a fructan that has high structural flexibility and good biodegradability, which allows for its use as a drug delivery system. In a study comparing chitosan with inulin for drug delivery, the small inulin NPs showed less toxicity and a higher accumulation in the lymphatic nodes (Crecente-Campo et al., 2019).

### 3.3 Polymeric nanoparticles

The potential of polymeric NPs relies on their highly versatile structure, which can be altered depending on the therapeutic application, cargo, or type of administration. Moreover, the chemical reactivity of the polymers can be exploited for the controlled release of drugs at different pH or thermal environments. These properties in addition to good biocompatibility, make polymeric NPs great candidates in the biomedicine field (Table 7) (Elsabahy and Wooley, 2012; Sartaj et al., 2021).

**TABLE 7:** Summary of polymeric NPs with their therapeutic area and functionalization strategy.

Polymer type	Purpose	Therapeutic area	Therapeutic Agent	Functionalization strategy	Ref.
Arginine based	Macrophages polarization from M2 to M1 profiles	Cancer	Toll-like receptor 3 agonist poly(I:C)	Poly(I:C) arginine-rich polypeptide was enveloped with an anionic polymeric layer by film hydration or incubation	Dacoba et al. (2020b)
Polyethylemine	Intratumoral immunotherapy			Nanoplexed formulation of Poly I:C complexed with polyethylenimine	Aznar et al. (2019)
Polyglutamic acid	Enhance the efficacy of first-line chemotherapeutics	Cancer (Triple negative breast cancer)	DOX	Nanogel particles were formed by Cu catalyzed azide-alkyne cycloaddition of polyglutamic acid and subsequently loaded with DOX	Duro-Castano et al. (2021a)
	Improve treatment of unresectable cancer by controlled release	Cancer	Gemcitabine	The low molecular weight nanogel N4-Octanoyl-2-deoxycytidine was loaded with hyaluronic acid and polyglutamic acid nanocapsules prepared by a self-emulsifying method	Staka et al. (2019)
	To develop zwitterionic pDNA delivery systems	-	Plasmid DNA delivery	Derivatization with oligoaminoamide residues for an efficient assembly containing five units of succinyl tetraethylene pentamine to develop a zwitterionic nonviral vectors	Nino-Pariente et al. (2017)
Polyglutamate	Develop multimodal NPs for AD	Neurodegenerative diseases	Bisdemethoxycurcumin or Genistein	80–100 nm sphere-like cross-linked self-assembled star-shaped Polyglutamic acid functionalized with Angiopep-2 to promote BBB permeation	Duro-Castano et al. (2021b)
Tert-Ser polyacetal	pH dependent and controlled release of drug	Cancer (prostate)	PTX	PTX was incorporated to the side-chains of the pH-susceptible and biodegradable tert-serinol polymer by a one-pot synthetic procedure	Fernandez et al. (2022)
Polyarginine	Delivery of two therapeutic agents for reverting MDSC-mediated immunosuppression	Cancer immunotherapy	RNAi and CCL2 chemokine	RNAs were associated to the nanocapsules through the RNA condensing capacity of the polyarginine shell. The chemokine was encapsulated in the aqueous domains of a glyceryl-monooleate (GMO)-based liquid-crystal core where	Ledo et al. (2019)
	miRNA delivery to the hippocampus	Neurodegenerative diseases	Specific miRNA mimic, miR-132	D-octaarginine was covalently conjugated to lauric acid and subsequently to RNA through electrostatic interactions of interest. Finally, the nanocomplexes were enveloped with protective polymers such as polyethyleneglycol - polyglutamic acid or hyaluronic acid to enhance stability and diffusion through the olfactory nasal mucosa	Samaridou et al. (2020)
	Oral delivery of insulin	Diabetes	Insulin	Polyarginine NPs were coated with PEGylated polyaminoacids to protect insulin	Niu et al. (2018)
Poly(N-vinyl caprolactam)	Develop microgels for drug delivery	Cancer	DOX	(PVCL)-based thermoresponsive microgels prepared by copolymerization of N-vinylcaprolactam monomer and ethylene glycol dimethacrylate stabilized by a reactive [poly(2-(acryloyloxy)ethyl]trimethylammonium chloride) cationic shell and loaded with DOX	Etchenausia et al. (2019)
Chitosan, polyarginine, and carboxymethyl-β-glucan	Improve targeting specific immune cells in the lymphatics	Vaccine development	-	Chitosan nanocapsulse were functionalized with positively charged (polyarginine) and negatively charged (carboxymethyl-beta-glucan) polymeric shells	Cordeiro et al. (2019)
Polyanionic carbosilane dendrimers	Inhibits the infection of human cytomegalovirus	Antiviral therapy		Polyanionic carbosilane dendrimers that present several sulfonate or sulfate groups in their periphery	Relano-Rodriguez et al. (2021)
Nanocapsules with a polymeric shell	Development of intranasal vaccination against *Mycobacterium tuberculosis*	Vaccine development	Imiquimod and a fusion protein formed by two antigens of *Mycobacterium tuberculosis*	Imiquimod was encapsulated in the oily core of the nanocapsules together with a fusion protein. Nanocapsules were functionalized with a polymer shell made of chitosan or inulin/polyarginine	Diego-Gonzalez et al. (2020)
Poly(β-amino esters)	Improve vaccine development		mRNA	mRNA NPs based on poly(beta aminoester) polymers	Fornaguera et al. (2021)
Poly(lactic-co-glycolic acid)	Improve BBB permeability and pharmacokinetics of compounds	Neurodegenerative diseases	PDE7 inhibitor	NPs were prepared in two different manners, by single emulsion and nanoprecipitation	Nozal et al. (2021)
			CDC7 inhibitor	Polymeric NPs were prepared by nanoprecipitation	Rojas-Prats et al. (2021)
	Enhance BBB penetration	Stroke	-	Functionalized with supramagnetic iron oxide NPs	Grayston et al. (2022)
	Improve antiretroviral therapy	HIV	HIV-1 peptide inhibitor	PLGA NPs were covered by glycol-chitosan to enhance delivery	Ariza-Saenz et al. (2017)
	Improve the delivery of bioactive peptides to inhibit HIV infection			PLGA NPs coated with glycol chitosan	Ariza-Saenz et al. (2018)
	Provide an efficient oral peptide administration	Diabetes	Hydrophobically modified insulin	Nanoemulsions and micelles formed by MPEG-2000-DSPE sodium tauocholate, Miglyol 812N and Polaxamer 407	Santalices et al. (2021)
	Improve pharmacokinetic properties of Licochalcone A	Ocular inflammation	Licochalcone A	PLGA was covalently bound to cell penetrating peptides through maleimide-PEG amine	Galindo et al. (2022)
	Deliver miRNA and atorvastatin simultaneously	Atherosclerosis	miRNA 124a and atorvastatin	Atorvastatin was loaded in PLGA NPs in the single emulsion synthesis, that were subsequently covered by chitosan to promote electrostatic interactions with the miRNA and finally functionalized with anti-VCAM and H4A3 antibodies	Leal et al. (2022)
	Improve tumor targeting	Cancer	Allyl-isothiocyanate	NPs were functionalized with an anti-EGFR antibody	Encinas-Basurto et al. (2018b)
1-vinylimidazole	Obtain a synergistic anti-inflammatory effect	Inflammation autoimmune diseases	Ketoprofen and dexamethasone	Copolymers were self-assembled by nanoprecipitation, and NPs presented a hydrophobic core formed by covalently linked ketoprofen and a hydrophilic shell mainly formed by vinylimidazole	Espinosa-Cano et al. (2020a), Espinosa-Cano et al. (2020b)
1-vinylimidazole and methacrylic derivative	Study the synergistic anti-inflammatory effect		Dexamethasone and naproxen	Polymeric NPs were self assembled incorporating covalently-linked naproxen and physically entrapped dexamethasone	Espinosa-Cano et al. (2020b)
CaCO_3_ core stabilized by poly(vinylsulfonic acid)	Optimize *in vivo* delivery specificity	Acute ischemic stroke	Thrombolytic serine protease	Layer-by-layer structure of CaCO_3_ cores later removed and stabilized by poly(vinylsulfonic acid). After macromolecule entrapment alternate layer of charged polyelectrolyte poly(sodium 4-styrenesulfonate) and poly(diallyldimethylammonium chloride) are added. Finally a coating with a layer of doped iron oxide NP or gelatin is performed	Correa-Paz et al. (2019)
Polyethylene and propylene glycol and ribose	Develop fuel-free propulsion NPs activatable by NIR	-	-	Micelles formed by polyethyleneglycol, polypropylenglycol copolymer and sodium oleate and then ribose was polymerized on the surface at high temperature yielding a bottle-structured particles	Xuan et al. (2018)

Given their versatility, several anti-cancer strategies have been accomplished. Aznar et al. have studied the immunotherapeutic profile of BO-112, a nanoplexed form of Poly I:C coupled to polyethylemine that prevents its degradation from proteases. Poly I:C is a synthetic analog of double stranded RNA that activates innate immune receptors and several formulations with different polymers have been tested in the clinic. BO-112 was locally injected, leading to the death of tumoral cells. Also, this nanoplexed Poly I:C showed an antitumoral activity through the induction of type I Interferon and CD8 T-cell infiltrates in the tumor (Aznar et al., 2019). These promising results motivated the development of two clinical trials of BO-112 in combination with antiPD-1 therapies or with pembrolizumab showing encouraging clinical benefits in cancer patients. Dacoba et al. developed an arginine-based poly (I:C) nanocomplex that induces the accumulation of endosomal toll-like receptor 3 agonists, which affect the polarization of the profile M2 to M1 (pro-inflammatory and antitumoral) in endosomal compartments. This strategy has been explored in cancer, where the polarization to M1 profile of tumor-associated macrophages could be a promising approach against tumors (Dacoba et al., 2020b). Another study was based on the study of intratumoral immunotherapy.

Poly amino acid nanogels are also an interesting strategy in the delivery of cancer treatment. Interestingly, these agents can be developed to release the cargo specifically in the tumor microenvironment as pH responsive particles (Arroyo-Crespo et al., 2018). Duro-Castano et al. engineered polyglutamic acid nanogels loaded with DOX as an effective strategy for the treatment of triple-negative breast cancer metastases, which effectively reduced lung and lymph node metastases (Duro-Castano et al., 2021a). In another strategy, Fernández et al. developed a tert-Ser polyacetal loaded with PTX forming NPs of 10–70 nm, which showed a controlled release of drug dependent on pH. The nanomedicine thus inhibited an early release and reduced primary tumors in animal models while also inhibiting metastasis (Fernandez et al., 2022).

Staka et al. developed a low molecular weight hydrogel formed by a nucleoside (N4-octanoyl-2-deoxycytidine). The gel accommodated multiple polymeric NPs loaded with gemcitabine, a chemotherapeutic drug. The gel released the encapsulated drug for a month, which could be used as a treatment for unresectable cancer (Staka et al., 2019).

Myeloid-derived suppressor cells are a target in adoptive T cells transfer. Ledo et al. developed multilayer polymer nanocapsules to co-deliver two drugs: RNAi polynucleotides and chemokine CCL2. These NPs may help modulate the activity of myeloid derived suppressor cells (Ledo et al., 2019).

Etchenausia et al. studied poly(N-vinyl caprolactam) (PVCL)-based thermoresponsive microgels with polymer brushes as potential drug delivery nanocarriers. These microgels were biocompatible on HeLa and RAW cells. They tested DOX-loaded microgels and determined a sustained release of DOX from microgels as well as increased cell viability compared to free DOX, confirming the suitability of these microgels as safe drug delivery nanocarriers (Etchenausia et al., 2019).

Cordeiro et al. designed synthetic and natural polymer NPs and nanocapsules for antigen delivery. They observed that small-size cationic nanoclusters showed high accumulation in the lymph nodes and concluded that by modifying the physicochemical properties and composition of the nanocapsules, modulation of lymphatic uptake and biodistribution would be possible (Cordeiro et al., 2019).

**FIGURE 6:**
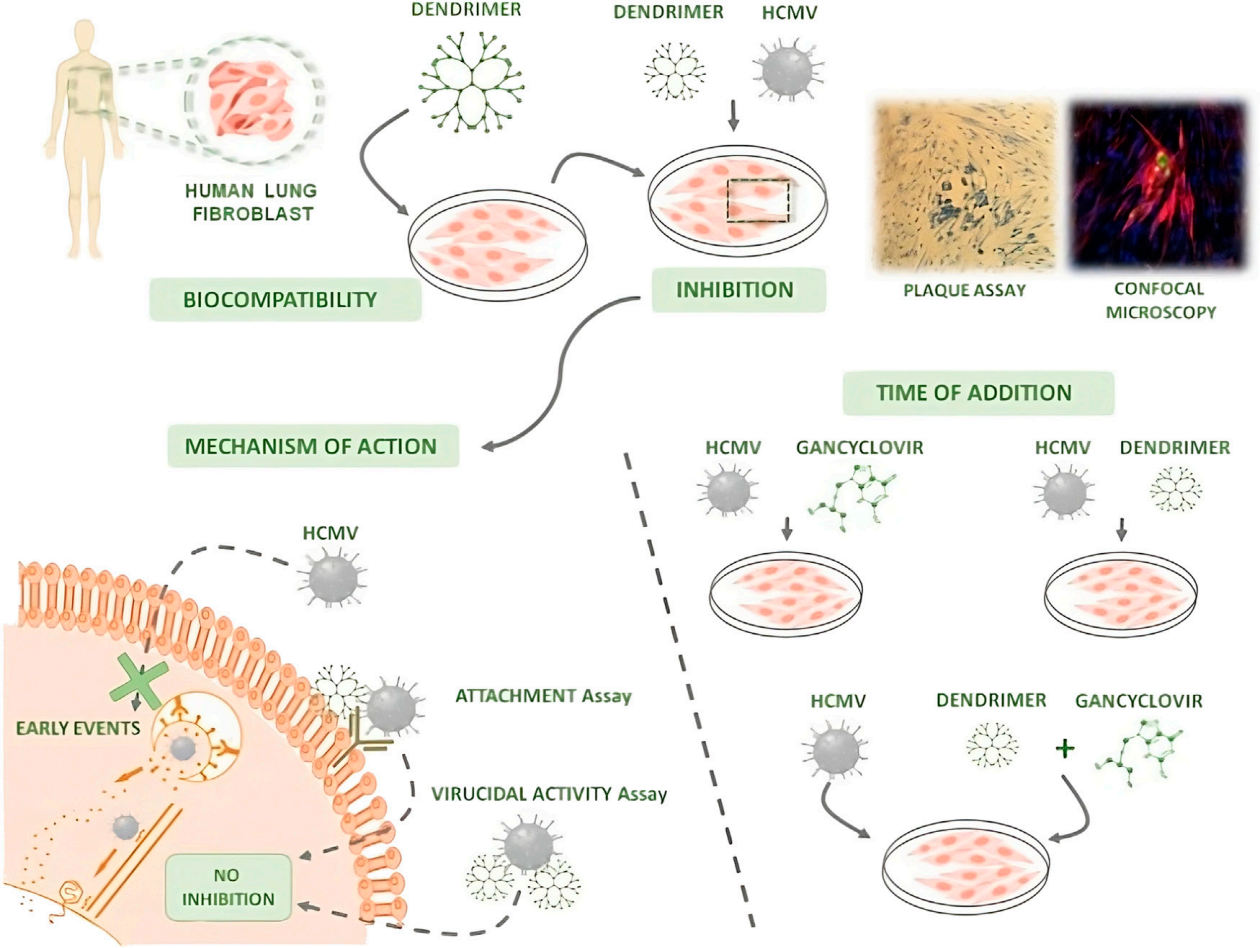
Dendrimer strategy as antiviral therapy. Reprinted from Relano-Rodriguez et al. (2021) and licensed under the Creative Commons Attribution.

Relano-Rodríguez et al. worked on human cytomegalovirus (HCMV), which infects and replicates in a wide variety of cells. They focused on the study of polyanionic carbosilane dendrimers (PCD). They tested two PCDs, G2-S16 and G2-S24P, which, alone or with current treatments, seemed to be a good tool against HCMV (Relano-Rodriguez et al., 2021) (Figure 6).

Polymeric nanocapsules are promising carriers for various antigens against different pathogens whose immunogenicity can be improved by including immunostimulatory molecules, improving current vaccines. Imiquimod (IMQ) has been described as a good modulator of innate immunity and activator of the Th1 immune response, which is the target of most vaccines. Previous work showed that encapsulation of IMQ in chitosan (CS) NC induced protective antibody levels against recombinant hepatitis B surface antigen (HB) in mice immunized intranasally. In this work, two different IMQ-loaded NPs with a CS or inulin/pArg polymeric shell were synthesized for the development of a model vaccine containing a recombinant fusion protein (RFP) derived from the ESAT-6 antigen and CFP-10 from Mtb. The vaccine containing INU:pArg:Ag nanocapsules was the most immunogenic prototype against the ECH fusion protein (Diego-Gonzalez et al., 2020). The use of mRNA in vaccination has achieved great success in preventing the acute effects of COVID-19 after SARS-Cov-2 infection. Fornaguera et al. developed and described a protocol for the preparation of poly(β-amino esters) and their conjugation to mRNA and studies in cellular models (Fornaguera et al., 2021).

Poly amino acid particles are also excellent carriers able to cross the BBB when conjugated to brain penetrant peptides, demonstrating interesting properties in treating central nervous system disorders such as Alzheimer’s disease (Duro-Castano et al., 2021b). Octaarginine conjugated with lauric acid forms a hydrophobic complex that can protect labile molecules by electrostatic and hydrophobic interactions, improving miRNA delivery in the hippocampus (Samaridou et al., 2020). Poly-I-glutamic acid complexes are also an efficient and non-toxic strategy to deliver oligonucleotides or DNA. Niño-Pariente et al. developed nanocomplexes with a poly-I-glutamic acid backbone that was derivatized with oligoaminoamide residues to efficiently assemble and deliver plasmid DNA (Nino-Pariente et al., 2017). Using PLGA-based polymeric NPs, Nozal et al., enhanced BBB permeation of NPs loaded with S14, a phosphodiesterase 7 (PDE7) inhibitor with great potential to treat Parkinson’s disease. According to the studies, S14-loaded PLGA-NPs showed improved pharmacokinetic properties of S14 in animal models, as well as enhanced safety of this inhibitor (Nozal et al., 2021). The same group also encapsulated PHA-767491, a potent cell division cycle 7 (CDC7) kinase inhibitor in PLGA-NPs improving its permeability and CNS delivery properties (Rojas-Prats et al., 2021).

Grayston et al. tested biocompatible PLGA nanocapsules functionalized with superparamagnetic iron oxide NPs and Cy7.5 to improve brain targeting of drugs after stroke. According to the results, the intra-arterial route for the cerebral administration of new treatments showed an extraordinary advantage (Grayston et al., 2022).

Another active area of research belongs to anti-inflammatory agents. Interleukin-12 and interleukin-23 have a high interest as therapeutic targets to treat autoimmune/inflammatory diseases and chronic inflammatory diseases where T cells are the primary cells that are dysfunctional. Espinosa-Cano et al. prepared anti-inflammatory polymeric NPs that combine ketoprofen and dexamethasone (14Dx-KT), which are one of the most efficient cyclooxygenase-inhibitors. According to the results, a long-term treatment with 14Dx-KT NPs reduced the expression of Il12b and IL23a to normal cellular levels. The results suggest that the ketoprofen-based systems present an anti-inflammatory activity reducing the basal levels of pro-inflammatory markers and increasing the gene expression of anti-inflammatory cytokines (Espinosa-Cano et al., 2020a). Recent studies demonstrate the anti-inflammatory capacity of dexamethasone and naproxen. However, their use is limited by the rapid clearance of free drug. Espinosa-Cano et al. used polymeric NPs to administer these drugs in order to accumulate in pathological tissue, increasing the drugs’ activity and reducing their adverse effects. The prepared naproxen-containing polymeric NPs loaded with dexamethasone were able to repress Il12b transcript levels, which would be an interesting treatment of autoimmune/anti-inflammatory diseases in which IL12 and IL23 are overexpressed (Espinosa-Cano et al., 2020b).

Oral delivery of insulin is also an attractive application for this type of NPs, since the acidity of intestinal fluids increases the risk of degradation. For instance, coating polyarginine insulin NPs with PEGylated polyaminoacids, protects insulin until it reaches the intestinal mucus (Niu et al., 2018), achieving the highest insulin uptake ever recorded in cellular models. Despite not resulting in a significantly increased systemic insulin uptake, the system showed a great potential for the delivery of peptides through the intestine mucosa. Santalices et al. also aimed at orally delivering hydrophobically modified insulin. They developed and extensively characterized nanoemulsions with selected components with improved properties on permeation, stability and mucodiffusion, such as miglyol, PEGylated phospholipids and poloxamer. The nanocomplexes showed promising results in cellular models and a moderate but significant hypoglycemic response in animals, highlighting key steps to take into consideration to overcome intestinal barriers (Santalices et al., 2021).

Similarly, Ariza-Saenz et al. used polymeric NPs of PLGA particles coated with glycol-chitosan (Ariza-Saenz et al., 2017) to enhance the delivery of a peptide inhibitor of the HIV-1 fusion protein that demonstrated to have enhanced permeability and efficacy of the peptide alone (Ariza-Saenz et al., 2018; Sanchez-Lopez et al., 2021).

As already showcased, polymeric NPs have been widely studied for the delivery of active compounds with poor pharmacokinetic properties. Here the delivery of licochalcone A was investigated using PLGA NPs and cell-penetrating peptides Tet and B6. Galindo et al. showed how the nanoconjugates with B6 showed increased activity for the treatment of ocular inflammation (Galindo et al., 2022).

Since combining drugs with miRNAs within a nanocarrier is a promising treatment for atherosclerosis, Leal et al., have developed polymeric PLGA NPs that simultaneously encapsulate and deliver miRNA-124a and the statin atorvastatin (ATOR). This combination reduced levels of proinflammatory cytokines and ROS. In addition, the dual-loaded NPs proved to be non-toxic to cells and prevent the accumulation of low-density lipoproteins inside macrophages and morphological changes, showing promise as a treatment for these types of diseases (Leal et al., 2022).

Encinas-Basurto et al., have developed allyl-isothiocyanate (AITC)-loaded PLGA NPs that target epithelial carcinoma cells due to anti-EGFR antibody binding to the surface. These NPs showed better anti-cancer properties compared to the free drug, suggesting that receptor-ligand binding could be used to target the NPs to tumor cells for improved drug delivery (Encinas-Basurto et al., 2018b).

In another attempt to generate smart-delivery NPs, Correa-Paz et al. engineered polymeric templated CaCO_3_ NPs with a layer-by-layer structure. The NPs were loaded with a fragile thrombolytic serine protease, labelled fluorescently and tagged with iron oxide NPs. The particles proved to efficiently release their protease cargo upon ultrasound application in cellular and animal models and maintained the activity after its delivery (Correa-Paz et al., 2019).

In another example of smart drug delivery using nanomaterials, Xuan et al. developed nanobottles formed from polyethylene glycol and ribose with a soft-template-based polymerization methodology. Using NIR light, the inner fluid of the bottle can be heated, resulting in its propulsion. The authors studied the trajectories, velocity and explosion events of the motors that could be controlled by modulating the NIR source and analyzed them through finite element analysis. This work is paving the road for the discovery and synthesis of new fuel-free based nanomotors (Xuan et al., 2018).

### 3.4 Lipid nanoparticles

Lipid NPs (LNPs) range from liposomes to solid LNPs (SLNs) and quatsomes (QS). The potential of these formulations relies on their stable structure and ability to cross biological barriers. LNPs constitute a good vehicle to transport both hydrophobic and hydrophilic drugs, providing protection for its cargo. For example, the complexation of lipids with nucleic acids allows their transport and prevents their degradation. They can be divided into four major types: liposomes, niosomes, SLNs, and nanostructured lipid carriers (NLCs) (Dhiman et al., 2021). SLNs and NLCs overcome liposomes and niosome due to their better performance under pH- and enzyme-dependent degradation. All of these formulations have been of research interest in Spain (Table 8) (Vargas-Nadal et al., 2020), (Tenchov et al., 2021)

**TABLE 8:** Summary of LNPs with their therapeutic area and functionalization strategy.

NP Type	Purpose	Therapeutic area	Therapeutic Agent	Functionalization strategy	Ref.
Solid lipid NPs (SLNs)	Edelfosine oral administration	Cancer	Edelfosine	NPs prepared with the homogenization and ultrasonication method	Gonzalez-Fernandez et al. (2018)
	Biodistribution study of edelfosine oral, intravenous and intraperitoneal administration			Edelfosine-LNS labeled with Technetium-99m	Lasa-Saracibar et al. (2022)
	Maslinic acid oral administration		Maslinic acid	Poloxamer407 (PMA), dicarboxylic acid-Poloxamer407 (PCMA) or Hialuronic Acid (Hyaluronic acid)-coated PCMA shell	Aguilera-Garrido et al. (2022)
	Improve the efficiency and the specificity of the SLN-loaded drug		Trans retinoic acid	SLNs composed of stearic acid, Epikuron 200 and sodium taurodeoxycholate coated with PE-PEG	Arana et al. (2019)
	Pulmonary administration	Tuberculosis	Rifabutin	RFB-loaded SLN based on glyceryl debehenate or glyceril tristearate	Gaspar et al. (2017)
	Drug administration	Tuberculosis	Bedaquiline	Chitosan-based nanocapsules with PEG layer	De Matteis et al. (2018)
	Local administration	Hearing loss	Dexamethasone and hydrocortisone	Stearic acid-based SLNs loaded with glucocorticoids	Cervantes et al. (2019)
	Oral drug delivery	Inflammatory bowel disease	Polyphenol oleuropein (OLE)	Olive oil-based NLCs	Huguet-Casquero et al. (2020)
		Antiparasitic (*Leishmania*)	Ammonium iodide derivative C6I	Glycerol tripalmitate and glyrerol tristearate SLNs obtained by emulsión-solvent evaporation method	Fernández et al. (2021)
	Nucleic acid delivery	-	Circular DNA and linear RNA	Combination of cationic and ionizable lipids	Fabregas et al. (2017)
			mRNA and pDNA	Combination of ionizable and cationic lipids (DOTAP)	Gomez-Aguado et al. (2020)
	Ocular drug delivery	Keratoconus	Lactoferrin	Double emulsion/solvent evaporation method	Varela-Fernandez et al. (2022)
	Gene therapy	Eye disease	IL-10	SLNs combined con protamine, dextran or hyaluronic acid and formulated with PVA.	Vicente-Pascual et al. (2020)
	Production of IL-10 in corneal cells		mRNA-based nanomedicinal products	Eye drops containing mRNA formulated in SLNs	Gomez-Aguado et al. (2021)
	Nucleic acid delivery (gene-silencing therapy)	-	siRNA	Cholesterol derivative cholesteryl oleate SLNs	Sune-Pou et al. (2018)
	Liver lipopolysaccharide-binding protein (LBP) downregulation	Obesity	Modified LBP siRNA	Four different lipid combination that interact with RNA and form non-charged NPs	Latorre et al. (2022)
	Downregulate metalloproteinase 9 (MPP-9)	Eye disease	Short-hairpin RNA (shRNA)	Nanocarriers formed by protamine, dextran and plasmids	Torrecilla et al. (2019)
Nanostructured lipid carriers (NLCs)	Characterization of IMT-loaded NLCs	Gastrointestinal stromal tumors	Imatinib	NLCs containing imatinib by emulsification-sonication methods	Gundogdu et al. (2022)
	Develop, characterize and assay Tripalm-NPs-PTX	Breast and Lung cancer	PTX	Glycerin tripalmitate NLCs loaded with PTX	Leiva et al. (2017)
	Induce cancer cell death by apoptosis	Cancer	Apo2L/TRAIL	Binding TRAIL on a lipid nanoparticle surface	Gallego-Lleyda et al. (2018)
	Provide effective NLCs formulations for intramuscular or intraperitoneal administration	Resistant bacteria	Sodium colistumethate (SCM) and amikacin (AMK)	NLC formulations using trehalose and dextran as cryoprotectants and positive charged chitosan as coating	Vairo et al. (2020)
	Compare the efficacy of NLC-colistin vs. free colistin	Colistin-resistant *Pseudomonas aeruginosa* biofilm	Colistin	Hot melt homogenization technique. Precirol®ATO 5 and Miglyol 812 core mixed with colistin sulfate	Sans-Serramitjana et al. (2017a)
Nanostructured lipid carriers (NLCs)	Develop safer cationic NLCs using machine learning algorithms	Glioblastoma	Atorvastatin Coumarin	Glycerol based lipids NLCs produced by high-shear homogenization-ultrasonication	Basso et al. (2021)
	Drug oral administration	Antiparasitic (*Leishmania*)	Diselenide	Glyceryl palmitostearate and diethylene glycol monoethyl ether-based NLCs loaded with Diselenide	Etxebeste-Mitxeltorena et al. (2021)
	Increase EGFR gene expression	Cell culture and tissue engineering	Recombinant human epithelial growth factor (rhEGF)	NLCs prepared by hot melt homogenization	Chato-Astrain et al. (2021)
	Pulmonary and intramuscular administration	Respiratory infections	Sodium colistimethate		Pastor et al. (2019)
	Drug delivery across BBB	Neurodegenerative diseases	Growth factors	Polyunsaturated fatty acids-based NLC modulated with chitosan and TAT	Hernando et al. (2022)
Liposomes	Increase the granulysin concentration at the site of contact with the target cell	Cancer	Granulysin	Binding granulysin to the LNP surface through the complex formed by histidine tail of the protein and Ni^2+^ of a quelant lipid	Soler-Agesta et al. (2022)
Quatsomes and liposomes	Delivery	Fabry disease	α-galactosidase A	Incorporation of the cationic miristalkonium chloride (MKC) surfactant to nanoformulations	Tomsen-Melero et al. (2021)
Quatsomes	Parenteral administration	Cancer	Myristalkonium chloride (MKC) and cholesterol	Quatsomes prepared by depressurization on an expanded liquid organic solution-suspension method	Vargas-Nadal et al. (2020)
	Drug delivery	Chagas disease	Benznidazole	Quatsomes and LNPs prepared using CO_2_ in a one-step procedure. Cyclodextrins by antisolvent precipitation	Vinuesa et al. (2017)
	miRNAs delivery	Cancer	miRNAs	Quatsomes composed of Chol and/or DC-Chol and quaternary ammonium surfactants	Boloix et al. (2022)
Lipoplexes	Cellular transfection		siRNA	Histidine-based gemini cationic lipids	Sanchez-Arribas et al. (2020a)
				Double-chain cationic lipid based on the argnine	Sanchez-Arribas et al. (2020b)
	pDNA delivery	-	pDNA	Mixture of a Gemini-Bolaamphiphilic Hybrid Lipid and DOPE	Martinez-Negro et al. (2018)

**FIGURE 7:**
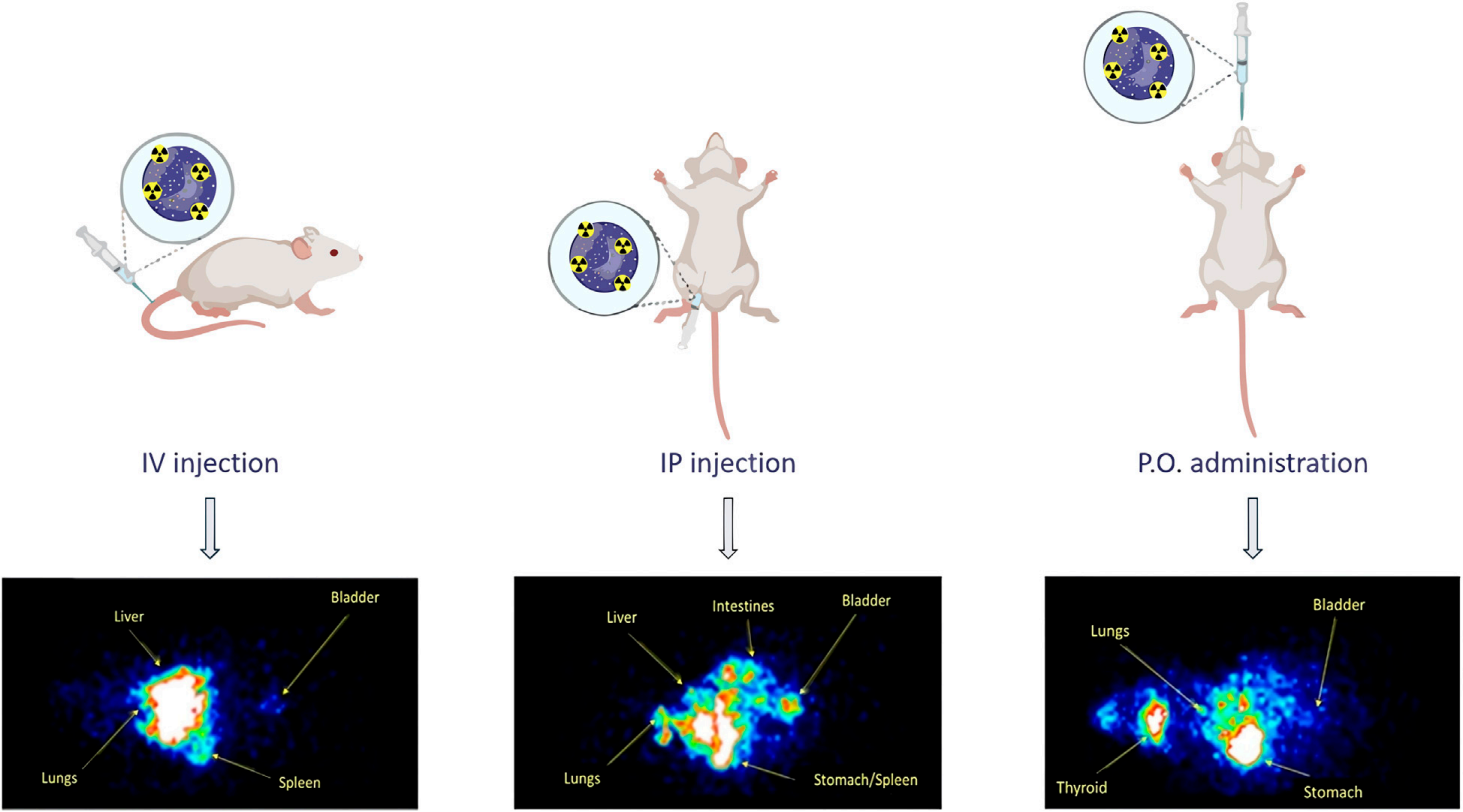
Distribution of LNPs loaded with edelfosine. Reprinted from Lasa-Saracibar et al. (2022), and licensed under the CReative Commons Attribution.

LNPs are vastly used to treat cancer. Mitxelena-Iribarren et al. tested the effectiveness of nanoencapsulated methotrexate against human bone osteosarcoma cells U2OS using microfluidic platforms that allow cell culture and incubation under highly controlled dynamic conditions (Mitxelena-Iribarren et al., 2017; Mitxelena-Iribarren et al., 2021). Cacicedo et al. combined bacterial cellulose hydrogel (BC) and NLC, including cationic or neutral DOX as a drug model and tested in human breast adenocarcinoma MDA-MB-231 cells and orthotopic breast cancer mouse model. These carriers showed a significant reduction in tumor growth, metastasis incidence and local drug toxicities (Cacicedo et al., 2018). González-Fernández et al. tested the oral administration of edelfosine encapsulated SLNs in osteosarcoma cancer cell lines and animal models. They found that oral administration had a better effect against primary osteosarcoma tumors and successfully prevented the metastatic spread of cancer cells from the primary tumor to the lungs. In addition, Lasa-Saracíbar et al. labelled these SLNs with Tc and studied their biodistribution in mice after intraperitoneal and intravenous administration. Results showed that the drug could reach circulation and provide a more constant blood concentration after intraperitoneal administration (Gonzalez-Fernandez et al., 2018; Lasa-Saracibar et al., 2022) (Figure 7). Aguilera-Garrido et al. tested the delivery maslinic acid using SLNs with three different shell compositions: Poloxamer 407 (PMA), dicarboxylic acid-Poloxamer 407 (PCMA), and Hyaluronic acid-coated PCMA (PCMA-HA) in Caco-2/HT29-MTX co-cultures. Interestingly, they found that the SLNPs improved the solubility of MA up to 7.5 mg/mL, stable in a wide range of pH, and increased the bioaccessibility of MA after gastrointestinal digestion in a cellular model (Aguilera-Garrido et al., 2022). Arana et al. studied how different amounts of phosphatidylethanolamine polyethylene glycol (PE⁻PEG) influence SLNs composed of stearic acid, Epikuron 200, and sodium taurodeoxycholate. They observed that the presence of PE⁻PEG improved active cell internalization of the NPs in an oral adenocarcinoma cell line, reducing non-specific internalization mechanisms. Furthermore, they also tested the effect of surface coating on the efficiency of incorporated drugs finding that PE⁻PEG coated SLN increases its chemotoxic effect compared to non-coated SL (Arana et al., 2019). Gundogdu et al. rigorously studied the physical characteristics of imatinib-containing NLC for the treatment of gastrointestinal stroma tumors. They found that these NPs revealed a Korsmeyer-Peppas drug release model of 53% at 8 h with above 90% of cell viability. They also found an IC_50_ of 23.61 μM and induction of apoptosis in CRL-1739 cell lines (Gundogdu et al., 2022). Leiva et al. tested glyceryl tripalmitate NLCs loaded with PTX. The NPs-PTX significantly enhanced PTX antitumor activity in human breast (MCF7, MDAMB231, SKBR3, and T47D) and lung (A549, NCI-H520, and NCI-H460) cancer cells. They also decreased the volume of breast and lung multicellular tumor spheroids (Leiva et al., 2017).

To induce apoptosis in cancer cells, Gallego-Leyda et al. employed SLNs decorated with TNF-related apoptosis-inducing ligand (TRAIL). They found that the decorated NPs were more cytotoxic than soluble TRAIL in A673 cells, RD cells and HT-1080 cells (Gallego-Lleyda et al., 2018). Similarly, Solar-Agesta et al. developed LNPs whose surfaces can bind granulysin as an antitumoral treatment, increasing the concentration at the site of contact with the target cell. Granulysin binding to the liposomes significantly increased the cytotoxic potency, produced mainly by apoptosis (Soler-Agesta et al., 2022).

NLC can overcome the toxicity of some antibiotics with a current limited use. Vairo et al. encapsulated antibiotics in different NLC formulations through high pressure homogenization, and showed that negatively charged SCM-NLC, with trehalose as cryoprotectant, had the best efficacy in several bacteria strains (Vairo et al., 2020). With a similar aim, Vinuesa et al. explored different lipid nanoformulation options for benznidazole, a commercially available drug currently used for the treatment of Chagas disease with a high toxicity, finding balanced conjugates of activity/toxicity (Vinuesa et al., 2017).

LNPs are also used to destroy biofilms. Sans-Serramitjana et al. studied the viability of *P. aeruginosa* biofilms treated with both free and nanoencapsulated colistin finding a more rapid killing of *P. aeruginosa* bacterial biofilms by nanostructure lipid carrier-colistin than by free colistin (Sans-Serramitjana et al., 2017a). The same group investigated the encapsulation of Tobramycin and its effect on planktonic and biofilm forms of *Pseudomonas*. They found that the nanoencapsulation of tobramycin did not improve its efficacy against planktonic *P. aeruginosa* but did improve its ability to eradicate *P. aeruginosa* biofilms (Sans-Serramitjana et al., 2017b).

Machine learning algorithms can be used to unravel hidden patterns in NP. In this work by Basso et al., the effects of two novel glycerol-based lipids, GLY1 and GLY2, on the architecture and performance of NLC were evaluated. Results showed that GLY1 circumvents the intrinsic cytotoxicity, is effective at increasing glioblastoma uptake, and exhibits encouraging anticancer activity (Basso et al., 2021).

Diselenide loaded in NLCs were developed by Etxebeste-Mitxeltorena et al. to treat visceral leishmaniasis. In this work, Diselenide (2m), a trypanothione reductase inhibitor, was loaded in glyceryl palmitostearate and diethylene glycol monoethyl ether-based NLCs. They found that diselenide 2m-NLCs drastically enhanced its intestinal permeability and provided plasmatic levels higher than its effective concentration (IC_50_). In *Leishmania infantum*-infected BALB/c mice, 2m-NLC reduced the parasite burden in the spleen, liver, and bone marrow by at least 95% after 5 doses (Etxebeste-Mitxeltorena et al., 2021).

SLNs can also be included in microspheres of appropriate size through a spray-drying technique. Gaspar et al. encapsulated SLNs containing rifabutin (pulmonary antibiotic) and tested their antimycobacterial activity in a murine model of infection with *Mycobacterium tuberculosis* (Gaspar et al., 2017). Moreover, tuberculosis was also treated using Bedaquiline encapsulation in SLNs and chitosan nanocapsules in a work performed by Matteis et al. The authors encapsulated this drug and found no cytotoxicity at the concentration needed to kill bacteria, (De Matteis et al., 2018), highlighting the great potential of this approach (Baranyai et al., 2021).

Another interesting application of SLNs was developed by Cervantes et al. to protect auditory cells from cisplatin-induced ototoxicity. SLNs were loaded with glucocorticoids (dexamethasone and hydrocortisone) and they were efficiently incorporated by auditory HEI-OC1. Their results showed that the encapsulation in SLNs increased the protective effect of low-doses of hydrocortisone and lengthened the survival of HEI-OC1 cells treated with cisplatin (Cervantes et al., 2019).

NLCs were also used to treat inflammatory bowel disease. Huguet-Casquero et al. loaded NLCs with polyphenol (antioxidant/antiinflammatory) oleuropein (OLE). NLC-OLE showed to be more effective in decreasing the TNF-α secretion and intracellular ROS by activated macrophages (J774) compared to the conventional form of OLE in a murine model (Huguet-Casquero et al., 2020)

Fernandez et al. developed a SLN to deliver N-iodomethyl-N, N-Dimethyl-N-(6,6-diphenylhex-5-en-1-yl) ammonium iodide (C6I). The SLNs were obtained by emulsion–solvent evaporation method. They were made of glycerol tripalmitate and glyceryl tristearate. They compared their performance with PLGA NPs. Although SLNs performed better than the free C6I, the SLNs were not capable of overcoming the performance of PLGA (Fernández et al., 2021).

Oligonucleotides are one of the biomolecules that reach their site of action less effectively. Thus, Fabregas et al. characterized the main physicochemical characteristics and binding capabilities of SLNs to oligonucleotides. They optimized the formulation of the NPs to efficiently transfect circular DNA and linear RNA molecules into cells (Fabregas et al., 2017). Gomez-Aguado et al. developed different SLNs by the combination of cationic and ionizable lipids, to deliver mRNA and pDNA. They evaluated their performance in human retinal pigment epithelial cells (ARPE-19) and human embryonic kidney cells (HEK-293). The results showed that SLNs containing only DOTAP (1,2-dioleoyl-3-trimethylammonium-propane) were the most promising formulations for nucleic acid delivery (Gomez-Aguado et al., 2020).

Similarly, Sanchez-Arribas et al. developed a strategy for siRNA delivery using cationic lipoplexes (Sanchez-Arribas et al., 2020a) enabling moderate to high gene lockdown levels. The authors also characterized the protein corona formed around these lipoplexes, that could have an influence on the silencing activity of these agents (Sanchez-Arribas et al., 2020b). Delivery of siRNAs was also performed by Suñé-Pou et al. The authors incorporated the cholesterol derivative cholesteryl oleate to produce SLN–nucleic acid complexes with reduced cytotoxicity and more efficient cellular uptake. They found that intermediate concentrations of cholesteryl oleate exhibited good stability and spherical structures with no aggregation (Sune-Pou et al., 2018). Latorre J. et al. generated a LNP containing a siRNA with a chemically modified lipopolysaccharide-binding protein in order to reduce the levels of fat accumulated in the liver. The NPs showed to be effective *in vivo,* stressing the potential of this therapy against fatty liver disease (Latorre et al., 2022). In another work, Torrecillas et al. developed a SLN-based shRNA delivery system. This system was designed to downregulate metalloproteinase 9 (MMP-9), a proangiogenic factor, in corneal cells for the treatment of corneal neovascularization associated with inflammation. The non-viral vectors based on SLNs were able to downregulate the MMP-9 expression in HCE-2 cells via gene silencing, and, consequently, to inhibit cell migration and tube formation (Torrecilla et al., 2019).

Martinez-Negro et al. developed a multidisciplinary approach using a nanocarrier built with gemini-bolaamphiphilic hybrid lipids. This strategy resulted in a non-cytotoxic delivery of DNA plasmids in cellular models (Martinez-Negro et al., 2018). With the same goal, nanovesicles have proven to be effective carriers of miRNA in a recent work from Bloix et al. They engineered quatsomes, for the delivery of miRNAs and other small RNAs into the cytosol of tumor cells. The miRNAs delivered by this methodology were able to reach their target destination and thus providing a potential platform for the delivery of these molecules (Boloix et al., 2022).

LNPs have also been employed to cross the BBB to deliver drugs to the brain. Hernando et al. developed polyunsaturated fatty acids (PUFA)-based NLCs, namely, DHAH-NLC. The carriers were modulated with BBB-permeating compounds such as CS and trans-activating transcriptional activator (TAT) from HIV-1. They quantitatively assessed the permeability of DHAH-NLCs in endothelial cells (BMECs). Successfully, they reported that TAT-functionalized DHAH-NLCs successfully crossed the BBB in a cellular model (Hernando et al., 2022).

Improving ocular drug delivery is a milestone that can greatly impact the treatment of eye disorders. Varela-Fernandez et al. designed, developed and performed the physicochemical characterization of lactoferrin-loaded NLCs as a new therapeutic alternative for the keratoconus treatment. Based on the preclinical base obtained, they concluded that NLCs were stable, non-toxic and showed mucoadhesive properties (Varela-Fernandez et al., 2022). Vicente-Pascal et al. designed SLN-based eye drops as gene delivery system to induce the expression of interleukin 10 (IL-10). Two kinds of SLNs combined with different ligands (protamine, dextran, or hyaluronic acid) and formulated with polyvinyl alcohol (PVA) were prepared and tested in cellular and animal models. SLN-based vectors were capable of transfecting corneal epithelial cells. Animal model experiments show that IL-10 could reach even the endothelial layer (Vicente-Pascual et al., 2020; Gomez-Aguado et al., 2021).

To generate human keratinocyte primary cell cultures with potential in tissue engineering Chato-Astrain et al. developed EGF-loaded NLCs. After testing in skin keratinocytes and cornea epithelial cells, and in two epithelial cancer cell lines, gene expression analysis showed that the NLCs were able to increase EGFR gene expression (Chato-Astrain et al., 2021). Tomsen-Melero et al. investigated the potential of nanovesicles as carriers of α-galactosidase A, specifically quatsomes and hybrid liposomes. These structures provided improved colloidal stability in comparison to nanoliposomes and the conditions to preserve α-galactosidase A activity were also characterized, thus resulting in promising nanostructures for the delivery of this enzyme (Tomsen-Melero et al., 2021). In another study, MKC-Quatsomes formed by cholesterol and myristalkonium chloride were highly stable in different media and remained unaltered in human plasma for 24 h. After studying their biodistribution in a xenograft colorectal model they accumulated in tumors, liver, spleen, and kidneys (Vargas-Nadal et al., 2020). In fact, given their stability, they have also been used to efficiently incorporate different fluorophores (Ardizzone et al., 2018a), including diketopyrrolopyrroles, which promoted their photophysical properties and were used for imaging experiments in Saos-2 osteosarcoma cell line (Ardizzone et al., 2018b).

Pasto et al. prepared sodium colistimethate-loaded NLCs to treat respiratory infections (multiresistant *P. aeroginosa*). Biodistribution assessments showed a mild systemic absorption after nebulization and a notorious absorption after intramuscular route (Pastor et al., 2019).

## 4 Perspective

In this review, we aimed to provide an overview of the nanomedical research currently being performed in Spain. We organized the literature based on NP type and the most common materials employed in their preparation. There is a large number of researchers devoted to engineering NPs with nanomedical applications, and although here we have only focused on NPs with therapeutic applications, there is also a substantial amount of scientists engineering NPs for diagnostic purposes (Ortgies et al., 2016; Jornet-Martinez et al., 2019; Portela et al., 2020). Similarly, the thorough study of NPs and their properties is fundamental to future applications of these therapeutic agents. Here, we have reviewed a fraction of the work, due to space limitations, on the more popular nanoformulations and their therapeutic applications (Table 9). It is important to highlight the diverse applications of every type of NPs, that showcase the versatility of these nanomedical materials and their potential (Alfranca et al., 2016; Perez-Hernandez et al., 2017; Vallet-Regi, 2022).

**TABLE 9:** Summary of NPs classified by therapeutic area.

Therapeutic area	Purpose	NP type	Agent	Main results	Ref.
Antiamoebical activity *(A. castellani*)	Develop of anti-amoebic contact lens solution	AgNPs	-	A significant increase in anti-amoebic activity was observed	Hendiger et al. (2021), Hendiger et al. (2020)
			Tannic acid	AgNPs conjugated with tannic acid showed potential as an anti-amoebic agent	Padzik et al. (2018)
Antimicrobial activity	Lactose-gated delivery	MSNs	Essential Oil cinnamaldehyde	Improved delivery by decreasing volatility of compound and increasing its local concentration	Poyatos-Racionero et al. (2021)
Antibacterial activity	Overcome biofilm barrier		Levofloxacin	Therapeutic efficacy of levofloxacin was increased	Martinez-Carmona et al. (2019)
		AuNR@MSN		NIR activated MSNs with photothermal and antimicrobial properties	García et al. (2021)
Antibacterial activity	Provide effective NLCs formulations for intramuscular or intraperitoneal administration against resistant bacteria	Nanostructured lipid carriers (NLCs)	Sodium colistumethate (SCM) and amikacin (AMK)	NLC showed equal activity to the free drug. Intraperitoneal administration was observed to be superior than the intramuscular route	Vairo et al. (2020)
Antibacterial activity (Colistin-resistant *P. aeruginosa* biofilm)	Compare the efficacy of NLC-colistin vs. free colistin	Nanostructured lipid carriers (NLCs)	Colistin	Time dependent study of biofim viability upon treatment, showing colistin-NLC a more rapid biofilm killing with similar cellular death activity to the free drug	Sans-Serramitjana et al. (2017a)
Antibacterial activity (osteomyelitis)	Improve therapeutic effect	MSNs	Moxifloxacin, rifampicin	NPs treatment prevented the premature release of the antibiotics and induced biofilm disaggregation	Aguilera-Correa et al. (2022a)
Antibacterial activity (tuberculosis)	Pulmonary administration	SLNs	Rifabutin	Biodistribution study confirmed that pulmonary administered rifabutin NPs reached organs in 15–30 min timeframe, exerting their therapeutic activity	Gaspar et al. (2017)
	Drug administration	SLNs	Bedaquiline	NP encapsulation reduced drug toxicity while maintaining therapeutic activity	De Matteis et al. (2018)
	Drug delivery	MSNs-AgBr and Ag@MSNs	-	Good antimycobacterial capacity of both types of NPs was found *in vitro*	Montalvo-Quiros et al. (2021)
Antibacterial activity (*E. coli* infection)	Treatment of *E. coli* infection	AuNPs		Inhibition of *E. coli* infection was observed after treatment with the biosurfactant stabilized NPs	Gomez-Grana et al. (2017)
Antibacterial activity (*K. pneumoniae*)	Drug delivery		Amikacin	The combination of AuNPs and the antiobiotic evidenced a synergistic effect representing a potential anti-bacterial nanomaterial	Aguilera-Correa et al. (2022b)
Antibacterial activity (*Campylobacter*)	Improve drug efficacy against multidrug resistant strains	GSH-Ag NPs	-	Results evidenced that these NPs are highly effective against the infection of multidrug resistant strains	Silvan et al. (2018)
Antibacterial activity (*S. aureus* and *E. coli*)	Treatment against bacterial infection by *S. aureus* and *E. coli*	Hybrid γ-Fe_2_O_3_/Ag nanocomposites		Synergistic properties between AgNPs and IONPs were found for the treatment of bacterial infection of *S. aureus* and *E. coli*	Luengo et al. (2020)
Antibacterial activity (*P. aeruginosa*)	Treatment against bacterial infection by *P. aeruginosa*	AgNPs	Probiotics	Combination of AgNPs with living probiotics showed anti-bacterial activity against *P. aeruginosa*	Sabio et al. (2021)
Antibacterial activity (peri-implantitis)	Prevention of implant associated infection (*S. aureus* and mixed oral bacteria flora infection)		-	NPs prevented the formation of bacterial biofilms proving their potential to be used in dental implants to prevent pre-implantitis	Perez-Tanoira et al. (2022)
Antibacterial activity (*S. aureus*)	Anti-biofilm treatment	AgNP@nanoMIL-125(Ti)NH_2_		Results showed a significant bacterial inhibition, proposing this composite as an active coating biofilm treatment	Arenas-Vivo et al. (2019)
Antibacterial activity (*S. aureus* and *P.aeruginosa*)	Improve antibiotic treatment of *S. aureus* and *P.aeruginosa* infection	AgNPs	Enzymes (α-amylase, cellulose, DNase I and proteinase K)	A potent anti-biofilm activity was found decreasing bacterial infection in combination with antibiotics	Rubio-Canalejas et al. (2022)
Antimicrobial activity (anti-fungal and anti-amoebic)	Treatment for fungal and amoebic infection	AgNRs and AgNws	-	AgNPs showed activity agains various fungals and *A. castellanii* infection	Gonzalez-Fernandez et al. (2022)
Antiparasitic (*Leishmania*)	Oral drug delivery	Solid lipid NPs (SLNs)	Ammonium iodide derivative C6I	Several NPs were tested in macrophages and PLGA based nanoformulationms improved antuiñlieshmanial activity on intracellular amastigotes	Fernández et al. (2021)
	Drug oral administration	Nanostructured lipid carriers (NLCs)	Diselenide	NLCs increased intestinal permeability providing higher plasmatic drug levels and reducing parasite burden after oral administration	Etxebeste-Mitxeltorena et al. (2021)
Chagas disease	Drug delivery	Quatsomes	Benznidazole	NPs enabled a safer administration of the drug without losing therapeutic activity	Vinuesa et al. (2017)
Antiviral activity (SARS-CoV-2)	Treat or prevent SARS-CoV-2 infection	Iron oxide NPs and iron oxyhydroxide NPs	**-**	IONPs and IOHNPs might be repurposed as a therapeutic treatment for SARS-CoV-2 infection	DeDiego et al. (2022)
Antiviral activity (HIV)	Enhance immune response in HIV infection	AuNPs	**-**	AuNPs coated with two different antibodies that recognize HIV protein and natural killer cells, promoting specific cell-to-cell contact and induced a potent cytotoxic response	Astorga-Gamaza et al. (2021)
	Drug delivery		siRNA-Nef	AuNPs were used for gene delivery against the HIV	Pena-Gonzalez et al. (2017)
	Improve antiretroviral therapy	Poly(lactic-co-glycolic acid)	HIV-1 Peptide inhibitor	NPs were successfully loaded with inhibitory peptides, permeated through the mucus reaching the vaginal epithelium and released the cargo	Ariza-Saenz et al. (2017)
	Improve the delivery of bioactive peptides to inhibit HIV infection			NPs successfully permeated vaginal tissue and released inhibitory peptides	Ariza-Saenz et al. (2018)
Antiviral therapy	Inhibits the infection of human cytomegalovirus	Polyanionic carbosilane dendrimers	-	Dendrimers reduced HCMV infection and enhanced the activity of ganciclovir	Relano-Rodriguez et al. (2021)
Cancer	Delivery	Multifunctional MSNs	DOX	Increase the efficacy of glucose dependent cargo delivery	Jimenez-Falcao et al. (2019)
		MSNs		A drug encapsulation system was designed using galacto-oligosaccharides to coat a silica scaffold containing the drug of interest, which is preferentially released in tissues with senescent cells	Munoz-Espin et al. (2018)
	Delivery	Nanovehicle MSNs	Camptothecin and DOX, Zn, and phtalocyanine	A pH-triggered nanovehicle with regioselectively bifunctionalized MSNs for the dual release of DOX and camptothecin was developed	Llinas et al. (2018)
		MCM-41/Pt	Ru(Bpy)_3_Cl_2_/DOX	Pt-MSNs nanomotors with stimuli-response drug release capabilities have been designed, synthetized and characterized	Diez et al. (2021)
		Au-MSNs Janus NPs	DOX	A nanodevice was designed for the autonomous release of DOX in specific cells triggered by NADPH and glutathione disulfide	Mayol et al. (2021)
	Synergy of hyperthermia and drug delivery	MSNs loaded with iron oxide NPs		The synergic effect of the intracellular hyperthermia and chemotherapy significantly reduced *in vivo* tumor growth without a global temperature rise of the tissue	Guisasola et al. (2018b)
	Photothermal therapy	Gold suprashells assembled around SPIONs	-	Development of multifunctional gold suprashell that can be magnetically accumulated and used for controlled plasmonic heat generation	Paterson et al. (2017)
	Delivery (drug nanocarrier)	Iron Oxide (magnetite) NPs	Crocin	Drug nanocarrier enhance therapy in comparison to free drug	Saravani et al. (2020)
	Magnetic hyperthermia (MHT) cytotoxicity		-	Cytotoxic effects caused by magnetic hyperthermia are reduced by the cell-promoted NP aggregation	Mejias et al. (2019)
	Cell internalization of IONPs			Magnetic thermal response can be predicted based on the type and size of the NP in the cellular media	Cabrera et al. (2018)
	Study of MHT in 3D cultures and a murine model			Localization of NPs inside or outside the cells can trigger different apoptotic routes. Best therapeutic conditions were tested in an animal model	Beola et al. (2018), Beola et al. (2020), Beola et al. (2021)
	To develop computational models to effectively design nanomedicine	Iron Oxide (maghemite) NPs	Gemcitabine	Rational design was successfully used to develop multifunctional NPs	Aires et al. (2017)
	Therasnotic agent	Au@Fe nanoflowers	-	Nanoflowers could be used as promising tools for diagnostics and hyperthermia therapy	Christou et al. (2022)
	MHT and NP uptake in cancer cells	Manganese ferrite nanoflowers	-	Improvement heating efficiency of magnetic NPs in a glioma cellular model	Del Sol-Fernandez et al. (2019)
	PTT, MHT and magneto-photothermal treatment	Gold-iron oxide Janus magnetic nanostars		A synergistic cytotoxic effect on cancer cells and in an animal model was observed	Espinosa et al. (2020)
	PTT, MHT and magneto-photothermal treatment	Gold coated magnetite nanorods		Proof of concept of the generation of free-standing anisotropic materials for magneto and photothermia applications	Rincon-Iglesias et al. (2022)
	Modulation of angiogenesis as an antitumor therapy	Super paramagnetic Iron oxide (maghemite) NPs		Anti-angiogenic and an-tumoral effects were observed after treatment with NPs	Mulens-Arias et al. (2019)
	Targeted cell therapy	Iron oxide (maghemite) NPs		Cell retention was favoured improving cell-based therapy for cancer treatment	Sanz-Ortega et al. (2019a)
	Targeted adoptive T-cell transfer therapy and EMF	Iron oxide NPs		T cells modified with the magnetic NPs were retained in lymph nodes after the use of an EMF despite their lower number	Sanz-Ortega et al. (2019b), Sanz-Ortega et al. (2019c)
	MHT *in vivo* feedback	Ag_2_S-based NPs, Fe_3_O_4_ MNPs		Therapeutic effect of the MNPs was more accurately evaluated	Ximendes et al. (2021)
Cancer	Drug delivery, controlled release and MHT	MNP@mSiO_2_	DOX	Drug was released at acidic pH showing promising results	Fuentes-García et al. (2021)
	Drug delivery, controlled release and MHT	MF66 iron oxide NPs		Drug release was controlled by pH reducing side effects of chemotherapy and increasing their therapeutic effect	Lazaro-Carrillo et al. (2020)
	Delivery	Iron oxide (maghemite) NPs	miRNA155, miRNA125b and miRNA146a	A pro-inflammatory response was induced after treatment with the loaded NPs	Lafuente-Gomez et al. (2022)
	Heterogeneous catalysis	Copper-iron oxide spinel NPs	-	NPs reduced the levels of glutathione and increased ROS and apoptotic pathways in cancer cells	Bonet-Aleta et al. (2022a), Bonet-Aleta et al. (2022b)
	Improve NP’s properties	AuNPs	-	NPs uptake was improved using an adenovirus	Gonzalez-Pastor et al. (2021)
	Drug delivery		Gemcitabine/Gapmers	Delivery of both agents reduced the chemoresistence to gemcitabine in cancer cells	Garcia-Garrido et al. (2021)
	Drug delivery	AuNPs	DOX	A synergetic effect between chemo and photothermal therapy was observed reducing cell viability in cancer cells	Encinas-Basurto et al. (2018a)
			Docetaxel/DOX	Combination of the chemotherapeutic properties of the drugs with the photothermal therapy of the NPs produced high cytotoxic effects in breast cancer cells	Villar-Alvarez et al. (2019)
			Doxycicline/Fosbretabulin	Co-delivery of the two drugs in combination with photothermal and photodynamic therapy showed promising results in an embryo xenograph model	Paris et al. (2020)
			DOX	Controlled release of the drug in combination with photothermal therapy evidenced a synergistic effect in cancer cells	Villar-Alvarez et al. (2018)
	Drug delivery	AuNPs	DOX	A nanosystem for the controlled release of the drug was developed	Martin-Saavedra et al. (2017)
			DOX/SN38	Controlled drug release of both drugs showed a potent anti-tumoral activity as well as anti-neoplastic activity against cancer stem cells	Latorre et al. (2019)
Cancer	Biosynthesis of AuNPs with therapeutic properties		-	NPs synthetized using an aqueous extract of *Saccorhiza polyschides* showed both, immunostimulant and anti-proliferative activities, on immune and tumor cells	Gonzalez-Ballesteros et al. (2021a)
	Green synthesis of AuNPs with therapeutic properties			NPs synthetized using extracted carrageenan from red seaweed presented anti-oxidant and anti-tumoral activities	Gonzalez-Ballesteros et al. (2021b)
	miRNA delivery	AuNPs	Natural killer cells extracellular vesicle-miRNA	Specific delivery of the NPs regulated the immune response representing a potential immunomodulatory approach for cancer treatment	Dosil et al. (2022)
	Drug delivery	AuNPs	DOX	The therapeutic agent triggered by NIR light showed a reduction in the viability of cancer cells	Hernandez Montoto et al. (2019)
	Drug delivery	PLGA@Ag_2_S and PLGA@Ag_2_S@SPION	Maslinic acid	An efficient encapsulation and controlled release of the therapeutic agent was observed in cellular models	Alvear-Jimenez et al. (2022)
		AgNPs and AuNPs	Raltitrexed	Drug modified NPs showed a strong inhibition on cancer cell viability	Morey et al. (2021)
	Drug delivery	Folate-targeted albumin-alginate NPs	PTX	Improved uptake of NPs by cells due to the overexpression of folate receptor	Martinez-Relimpio et al. (2021)
	Treatment of different types of tumors	AgNPs-LE AgNPs-PE	*A. muricata* extracts	Lower concentrations were found to have a potential antitumor activity with a better therapeutic index for the treatment of different types of tumors	Gonzalez-Pedroza et al. (2021)
	Macrophages polarization from M2 to M1 profiles	Arginine based	Toll-like receptor 3 agonist poly(I:C)	Nanocomplexes enabled a safe delivery of the Poly (i:C) that induced pro-inflammatory state macrophages	Dacoba et al. (2020b)
	Intratumoral immunotherapy	Polyethylenimine	Toll-like receptor 3 agonist poly(I:C)	Nanoplexed formulation of Poly I:C induced immunogenic cell death of tumoral cells	Aznar et al. (2019)
	Improve tumor targeting	Poly(lactic-co-glycolic acid)	Allyl-isothiocyanate	Antibody functionalized NP showed improved antitumoral properties than the free drug in cell co-cultures	Encinas-Basurto et al. (2018b)
	Develop microgels for drug delivery	Poly(N-vinyl caprolactam)	DOX	Sustained release of DOX through clathrin dependent internalization	Etchenausia et al. (2019)
	Improve treatment of unresectable cancer by controlled release	Polyglutamic acid	Gemcitabine	Lasting control release of loaded drug more than 1 month and effective cell growth inhibition in resistant cancer cell lines	Staka et al. (2019)
	Edelfosine oral administration	Solid lipid NPs (SLNs)	Edelfosine	NPs slowed down primary tumor growth and prevented metastatic spread. Combination with DOX did not show synergistic effects	Gonzalez-Fernandez et al. (2018)
	Biodistribution study of edelfosine oral, intravenous and intraperitoneal administration		Edelfosine	Biodistribution map of NPs after intravenous and intraperitounal administration showing that NPs presented quantifiable and constant levels in blood after intraperitoneal dose	Lasa-Saracibar et al. (2022)
Cancer	Maslinic acid oral administration		Maslinic acid	NPs improved solubility of the therapeutic agent, yielding delivery across *in vitro* gut barrier models, being able to inhibit the growth of pancreatic cancer cells	Aguilera-Garrido et al. (2022)
	Improve the efficiency and the specificity of the SLN-loaded drug		Trans retinoic acid	Phosphatidylethanolamine polyethylene glycol NPs improve active cellular internalization increasing the chemotoxic effect of the drug	Arana et al. (2019)
	Induce cancer cell death by apoptosis	Nanostructured lipid carriers (NLCs)	Apo2L/TRAIL	Nanoformulation development for apoptosis-inducing ligand with efficacy in sarcoma cell lines	Gallego-Lleyda et al. (2018)
	Increase the granulysin concentration at the site of contact with the target cell	Liposomes	Granulysin	Granulysin cytotoxicity is increased when formulated with liposomes and acts through the mitochondrial apoptotic pathway	Soler-Agesta et al. (2022)
	Parenteral administration	Quatsomes	Myristalkonium chloride (MKC) and cholesterol	Quatsomes are useful for biodistribution studies after intravenous administration and drug delivery applicability	Vargas-Nadal et al. (2020)
	miRNAs delivery		miRNAs	Quatsomes protect miRNA and provide a pH sensitive delivery platform	Boloix et al. (2022)
	Cellular transfection	Lipoplexes	siRNA	Remarkable silencing activity was obtained without associated toxicity	Sanchez-Arribas et al. (2020a)
				Cationic lipids with a helper lipid are a safe and biocompatible gene silencing strategy	Sanchez-Arribas et al. (2020b)
Cancer (Triple negative breast cancer)	Enhance the efficacy of first-line chemotherapeutics	Polyglutamic acid	DOX	Effective reduction of lung and lymph node metastases in triple-negative breast cancer	Duro-Castano et al. (2021a)
Cancer (prostate)	pH dependent and controlled release of drug	Tert-Ser polyacetal	PTX	Sustained release of PTX (2 weeks) reducing systemic toxicities while conserving tumor growth inhibitory activity	Fernandez et al. (2022)
Cancer immunotherapy	Delivery of two therapeutic agents for reverting MDSC-mediated immunosuppression	Polyarginine	RNAi and CCL2 chemokine	Nanocapsules modulated monocyte differentiation into tumour-associated macrophages and reduced significantly C/EBPβ mRNA levels	Ledo et al. (2019)
Cancer (pancreatic)	Development of targeted delivery chemotherapy	Iron oxide NPs	Gemcitabine and anti-CD47 antibody	Improved results and delivery were shown after treatment with the multifunctional NPs	Trabulo et al. (2017)
	MHT in mice model	Iron Oxide (maghemite) NPs	-	Temperature rise during MHT can be controlled by modulating the field intensity in animal models	Beola et al. (2021)
Cancer (sarcoma)	Microswimmers with MHT capacity	Manganese ferrite NPs	-	Microswimmers can be employed to enhance tissue penetration for specific cargo delivery	Ramos-Docampo et al. (2019)
Cancer (breast)	Enzyme prodrug therapy	AuNPs	Horseradish peroxidase	This combination strategy evidenced high anti-tumoral activity in 3D tumor models	Vivo-Llorca et al. (2022)
Breast and Lung cancer	Develop, characterize and assay Tripalm-NPs-PTX.	Nanostructured lipid carriers (NLCs)	PTX	PTX tumor activity was increased in breast and lung cancer cells through glyceryl triplamitate NPs formulation	Leiva et al. (2017)
Brain cancer	Hyperthermia control	AuNPs	-	Hybrid probes were internalized in 3D tumor spheroids and can induced cell death through photothermal effects, while measuring the local temperature *in situ*	Quintanilla et al. (2019)
Glioblastoma	Develop safer cationic NLCs using machine learning algorithms	Nanostructured lipid carriers (NLCs)	Atorvastatin Coumarin	Two novel glycerol lipids were studied showing that GLY1 circumvents the intrinsic cytotoxicity of common surfactant CTAB and shows anticancer activity	Basso et al. (2021)
Gastrointestinal stromal tumors	Characterization of IMT-loaded NLCs	Nanostructured lipid carriers (NLCs)	Imatinib	Imatinib loaded NPs were synthesized and characterized, showing a controlled release of the drug and thus being promising for tumor treatment	Gundogdu et al. (2022)
Eye disease	Gene therapy	Solid lipid NPs (SLNs)	IL-10	SLNs formulations were safe after topical administration and hyaluronic acid based ones reached endothelial layer	Vicente-Pascual et al. (2020)
	Production of IL-10 in corneal cells	Solid lipid NPs (SLNs)	mRNA-based nanomedicinal products	SLNs presented a high transfection efficiency when formulated as eye drops	Gomez-Aguado et al. (2021)
	Downregulate metalloproteinase 9 (MPP-9)	Solid lipid NPs (SLNs)	Short-hairpin RNA (shRNA)	NPs with non viral vectors downregulated MMP-9 expression in human corneal cells	Torrecilla et al. (2019)
Fabry disease	Delivery comparison by quatsomes and hybrid liposomes	Quatsomes and liposomes	α-galactosidase A	Improved efficacy was observed with hybrid liposomes having a good *in vitro*/*in vivo* safety profile	Tomsen-Melero et al. (2021)
Hearing loss	Local administration	Solid lipid NPs (SLNs)	Dexamethasone and hydrocortisone	Loaded NPs penetrated into auditory cells and protected them from cisplatin induced ototoxicity	Cervantes et al. (2019)
Inflammatory bowel disease	Oral drug delivery	Solid lipid NPs (SLNs)	Polyphenol oleuropein (OLE)	NPs ameliorated inflammation in macrophages and in a mouse model of acute colitis	Huguet-Casquero et al. (2020)
Keratoconus	Ocular drug delivery	Solid lipid NPs (SLNs)	Lactoferrin	Successful preparation of lactoferrin SLNs with a controlled release pattern	Varela-Fernandez et al. (2022)
Obesity	Liver lipopolysaccharide-binding protein (LBP) downregulation	Solid lipid NPs (SLNs)	Modified LBP siRNA	Nanoformulated siRNA against liver LBP is a promising therapy for faty liver associated to obesity	Latorre et al. (2022)
-	Increase EGFR gene expression	Nanostructured lipid carriers (NLCs)	Recombinant human epithelial growth factor (rhEGF)	NLCs improved EGF expression enhancing the efficiency of explant-based methodologies for primary cell culture	Chato-Astrain et al. (2021)
Respiratory diseases	Delivery	TNFR-Dex-MSN	Dexamethasone	A selective uptake by macrophages of the NPs was observed, demonstrating lung accumulation and reduction of the damage	Garcia-Fernandez et al. (2021a), Garcia-Fernandez et al. (2021b)
	Pulmonary and intramuscular administration	Nanostructured lipid carriers (NLCs)	Sodium colistimethate	NLC enabled a dose reduction of the drug to obtain a similar *in vivo* effect without apparent toxicity	Pastor et al. (2019)
Tissue regeneration (bone)	Delivery	MSN@PEI	Osteostatin and siRNA	Both therapeutic agents were efficiently delivered inside cells and consequently a synergistic effect in the increase in the osteogenic markers was observed	Mora-Raimundo et al. (2019)
	Induce osteogenesis and bone repair	MSNs	Ipriflavone	Results showed that both, bone regeneration and angiogenesis, were promoted after injection in an animal model	Arcos et al. (2022)
Tissue regeneration (angiogenesis)	Enhance angiogenesis			NPs evidenced a great potential to enhance angiogenesis after intracellular uptake	Casarrubios et al. (2021)
Tissue regeneration (periodontal)	Periodontal augmentation			NPs stimulated differentiation of pre-osteoblasts into mature osteoblasts after clathrin-dependent internalization	Casarrubios et al. (2020)
Vaccine development	Delivery	MSNs	Immunomodulatory and vesicle-associated proteins (Ag85B, LprG and LprA)	The designed nanosystems have been extensively characterized and their their immunostimulatory capacity demonstrated	Montalvo-Quiros et al. (2020)
	Improve targeting specific immune cells in the lymphatics	Chitosan, polyarginine, and carboxymethyl-β-glucan	-	Lymphatic uptake of polymeric NPs is dependent on particle size and charge	Cordeiro et al. (2019)
	Development of intranasal vaccination against *Mycobacterium tuberculosis*	Nanocapsules with a polymeric shell	Imiquimod and a fusion protein formed by two antigens of *Mycobacterium tuberculosis*	Inulin polyarginine produced an adequate IgA response *in vivo*	Diego-Gonzalez et al. (2020)
	Improve vaccine development	Poly(β-amino esters)	mRNA	Detailed description of a simple production of mRNA polymeric NPs with a proof of concept immunization	Fornaguera et al. (2021)
Neurodegenerative diseases	Develop multimodal NPs for AD	Polyglutamate	Bisdemethoxycurcumin or Genistein	Angiopep-2 conjugated NPs were efficiently delivered through the BBB and the treatment reduced β-amyloid peptides and rescued cognitive impairments in mice	Duro-Castano et al. (2021b)
	miRNA delivery to the hippocampus	Polyarginine	Specific miRNA mimic, miR-132	Successful production of a scalable nanoformulation that was efficiently delivered to the brain to exert its therapeutic action by nasal administration	Samaridou et al. (2020)
	Improve BBB permeability and pharmacokinetics of compounds	Poly(lactic-co-glycolic acid)	PDE7 inhibitor	Successful preparation of polymeric nanoparticle with efficient encapsulation and a sustained cargo release in mice brains after oral administration	Nozal et al. (2021)
			CDC7 inhibitor	Successful preparation of polymeric nanoparticle with efficient encapsulation and permeability through the BBB	Rojas-Prats et al. (2021)
	Drug delivery across BBB	Nanostructured lipid carriers (NLCs)	Growth factors	TAT-NLCs showed good BBB permeability *in vitro* and reduced the inflammatory response in human microglia	Hernando et al. (2022)
Diabetes	Oral delivery of insulin	Polyarginine	Insulin	Enhanced epithelial accumulation of insulin that did increase insulin transport	Niu et al. (2018)
	Provide an efficient oral peptide administration	Poly(lactic-co-glycolic acid)	Hydrophobically modified insulin	Nanoemulsions internalized Caco-2 monolayers yielding to a moderate hypoglycemic response in diabetic rats	Santalices et al. (2021)
Stroke	Enhance BBB penetration	Poly(lactic-co-glycolic acid)	-	Controlled brain delivery of polymeric NPs by endovascular administration and magnetic targeting	Grayston et al. (2022)
Ocular inflammaition	Improve pharmacokinetic properties of licochalcone A	Poly(lactic-co-glycolic acid)	Licochalcone A	Development of ocular inflammation targeted NPs with therapeutic efficacy *in vivo*	Galindo et al. (2022)
Atherosclerosis	Deliver miRNA and atorvastatin simultaneously	Poly(lactic-co-glycolic acid)	miRNA 124a and atorvastatin	NPs delivered simultaneously miRNA and atorvastatin that significantly reduced proinflammatory cytokines and prevented the accumulation of low-density lipoproteins inside macrophage	Leal et al. (2022)
Inflammation autoimmune diseases	Obtain a synergistic anti-inflammatory effect	1-vinylimidazole	Ketoprofen and dexamethasone	Synergistic anti-inflammatory effect of non-toxic polymeric NPs loaded with dexamethasone and naproxen. Coumarin loaded NPs showed to be rapidly uptaken by macrophages	Espinosa-Cano et al. (2020a)
	Study the synergistic anti-inflammatory effect	1-vinylimidazole and methacrylic derivative	Dexamethasone and naproxen		Espinosa-Cano et al. (2020b)
Acute ischemic stroke	Optimize *in vivo* delivery specificity	CaCO_3_ cores stabilized by poly(vinylsulfonic acid)	Thrombolytic serine protease	Encapsulation of therapeutic agent precluded its inactivation promoting blood clots breakdown *in vitro*. Delivery upon ultrasound application was confirmed *in vivo*	Correa-Paz et al. (2019)
-	To develop zwitterionic pDNA delivery systems	Polyglutamic acid	Plasmid DNA delivery	Successful plasmid DNA complexes formation that yielded an effective transfection in N2a cells without apparent toxicity	Nino-Pariente et al. (2017)
	Develop fuel-free propulsion NPs activatable by NIR	Polyethylene and propylene glycol and ribose	-	Succesful development of NIR light powered carbonaceous nanobottles	Xuan et al. (2018)
	Gene therapy	AuNPs	Plasmid DNA	A novel strategy to control the release of plasmid DNA after sternal stimuli as irradiation was developed	Sanchez-Arribas et al. (2021)
	Multi-Hot-Spot magnetic inductive nanoheating	Iron oxide (magnetite/maghemite) NPs	-	Creation of simultaneous and sequential multi hot spot conditions in a single pot	Ovejero et al. (2021b)
	Nucleic acid delivery	Solid lipid NPs (SLNs)	Circular DNA and linear RNA	Implementation of SLN formulation to efficiently transfect DNA and RNA	Fabregas et al. (2017)
	Nucleic acid delivery	Solid lipid NPs (SLNs)	mRNA and pDNA	Study of several SLNs for nucleic acid delivery analyzing stability and transfection efficieny	Gomez-Aguado et al. (2020)
	Nucleic acid delivery (gene-silencing therapy)	Solid lipid NPs (SLNs)	siRNA	Cholesteryl oleate SLNs represent a safe and efficient transfection tool for nonviral nucleic acid delivery	Sune-Pou et al. (2018)
	Increase the cellular attachment	AuNPs	-	Cellular adherence was increase enabling the thermoablating effect of AuNPs without cellular internalization	Artiga et al. (2018)
	Control protein corona formation			Protein corona formation was controlled and thus NPs cellular uptake increased	Mosquera et al. (2020)
	Inhibition of enzymatic activities			Local generation of plasmonic heat inhibited the horseradish peroxidase and the glucose oxidase which were coupled to AuNPs	Thompson et al. (2017)
	pDNA delivery	Lipoplexes	pDNA	Amphiphilic lipids and DOPE yieldied and efficient DNA transfection	Martinez-Negro et al. (2018)

Indubitably, one of the most explored abilities of NPs is their role as nanocarriers. In fact, in the last years the delivery function continues to improve, reaching a finely tuned controlled release that can be achieved through nanoformulation, and be sensitive to changes in the NPs’ environment, i.e., temperature light, ultrasounds or pH (Xuan et al., 2018; Diez et al., 2021; Mayol et al., 2021; Aguilera-Correa et al., 2022a). Exquisitely controlled layer-by-layer design of NPs enables to control and achieve drug release with spatiotemporal resolution, improving currently available therapeutic options (Correa-Paz et al., 2019). In addition, targeting key pathological cells present in several diseases such as senescent cells through their high β-galactosidase activity was achieved through galacto-oligosaccharides encapsulated drugs, yielding a versatile strategy potentially effective for multiple pathologies (Munoz-Espin et al., 2018). This delivery function can be further improved with the combination of self-propelled materials, (Ramos-Docampo et al., 2019), or synergistic delivery of therapeutic agents as demonstrated by the delivery of siRNA and a peptidic drug with remarkable potential against osteoporosis (Mora-Raimundo et al., 2019). The field however still shows important challenges ahead as highlighted in the aimed insulin oral delivery (Niu et al., 2018) that did not translate into an increased systemic absorption of insulin despite achieving a high retention in enterocytes.

The delivery of therapeutic agents and oligonucleotides based on polymeric nanocomplexes that stabilize and shield RNA analogs enabled the delivery of poly(I:C) to effectively treat several cancers. The preclinical data motivated clinical trials coordinated from Spain in which BO-112 was administered alone or in combinations treatments and performed in several hospitals with positive clinical benefits for some patients (Márquez-Rodas et al., 2022), (NCT: 02828098) (Therapeutics, 2016), (NCT: 05265650) (Clinica Universidad de Navarra UdN, 2022a), (NCT: 04570332) (Therapeutics, 2020). Interestingly, the same poly(I:C) agent in combination with polysaccharide based NPs has been employed for the development of another challenging therapeutic area: the creation of the HIV vaccine. Hyaluronic and chitosan NPs decorated with conserved HIV peptides as antigens and loaded with poly(I:C) yielded in a strong activation af antigen presenting cells (Dacoba et al., 2019).

Another clinical trial has its aim at evaluating antibiotic-containing alginate NPs for the treatment of *Pseudomonas aeruginosa* infection and biofilm formation in bronchiectasis patients. These type of NPs have shown to be efficient in treating pulmonary infections (Dhand, 2018).

Protein NPs also show promising characteristics for the delivery of insulin which yielded in clinical translation. Specifically the preclinical results obtained with Zein NPs has motivated the design of a clinical trial to verify in humans whether they could provide an efficient glycemic control in diabetic and obese patients through and orally administered therapeutic insulin derivative (NCT: 05560412) (Clinica Universidad de Navarra UdN, 2022b).

Regarding clinical applications, organic NPs and biodegradable MSNs have a competitive advantage over other metallic NPs. However, the remarkable properties that the metallic NPs have motivate their research for fighting complex diseases (Thompson et al., 2017). Thus, the combination of several cores in Janus–type NPs yields highly efficient conjugates that can not only combine complementary drugs or targeting agents but also synergistic effects simultaneously whith great potential to improve the treatment of several diseases (Lopez et al., 2017; Espinosa et al., 2020).

One of the hardest and more significant step in nanomedical development entails reaching clinical trials. This normally challenging road can have additional hurdles to achieve suitable nanoformulations. To translate nanomedicines into the clinic, multiple challenges should be taken into account to manufacture them under quality guidelines regulations (Foulkes et al., 2020). First, the results observed in research settings must be contextualized and standardized to prepare them for clinical translation. While small molecules are commonly evaluated for their efficacy, toxicity, and side effects, nanotherapies must also prove the biocompatibility for each of the components within the formulation. The use of specific nanomaterials for the development of personalized treatment requires in depth understanding of disease biology and the interaction between nanomaterials and the body. The potential of inorganic NPs in biomedicine is still in the preliminary stages of the clinic. Some important challenges in terms of biodistribution, pharmacokinetics, metabolism, biological barriers, safety, large-scale synthesis, patient heterogeneity or overall cost must be taken into account and optimized (Perez-Hernandez et al., 2017). In addition, several key parameters of the intrinsic NP-derived effects such as magnetic hyperthermia must be extensively studied and characterized to better understand its therapeutic operability and improve clinical translation (Beola et al., 2021; Luengo et al., 2022). On the objectives of the Spanish Scientific Network HIPERNANO stablished among different national groups was to consolidate the current scientific knowledge on hyperthermal therapies to enhance their practical implementation in clinical development (Spanish Scientific Network HiperNano, 2018). In addition, a key milestone was reached in 2022, where the first patient with pancreatic cancer was treated with magnetic hyperthermia in combination with the standard of care at Vall d’Hebron hospital (NoCanTher Project, 2022). This pilot study will guide future interventions and shed light on the steps needed to bring nanomedicines to the clinic. From this experience, it is worth highlighting that it is essential to involve multiple stakeholders, from scientists to clinicians, at every level (e.g., nanotherapy and clinical study design) from the beginning of the project to increase the success of this kind of study (Liz-Marzán et al., 2022). Furthermore collaborative nets and workshops such as NanomedSpain, Nanbiosis, Ciber-bbn and NanoSpain enable to promote collaborative studies with this goal.

Finally, while in this review we have only focused on the therapeutic role of NPs, there is another substantial part of nanomedicine that involves the diagnosis of diseases and in which an extensive amount of research groups in Spain are engaged. Both methods to diagnose *in vivo* (Ximendes et al., 2021), (Ximendes et al., 2017), (Pellico et al., 2021), (Santos et al., 2020) and *ex vivo* (Pelaez et al., 2018), (Litti et al., 2021), (Enshaei et al., 2021) enabled by several nanoformulations have revolutionized current medical practices and will continue to improve the crucial ability to diagnose diseases efficiently. In addition, the continuous effort of researchers to understand and exquisitely characterize the fate of NPs in biological system is a crucial foundation for all this work (Carregal-Romero et al., 2021; Areny-Balaguero et al., 2022).

## Footnotes

1 Hybrid here refers to nanostructures combining two types of inorganic materials in which both are clearly distinguishable (e.g., core-shell, aggregates/encapsulates of individual NPs of both materials) and both provide a relevant function for the application (i.e., are not used just as a coating or as a platform).
